# Ruthenium Complexes with Protic Ligands: Influence of the Position of OH Groups and π Expansion on Luminescence and Photocytotoxicity

**DOI:** 10.3390/ijms24065980

**Published:** 2023-03-22

**Authors:** Olaitan E. Oladipupo, Meredith C. Prescott, Emily R. Blevins, Jessica L. Gray, Colin G. Cameron, Fengrui Qu, Nicholas A. Ward, Abigail L. Pierce, Elizabeth R. Collinson, James Fletcher Hall, Seungjo Park, Yonghyun Kim, Sherri A. McFarland, Igor Fedin, Elizabeth T. Papish

**Affiliations:** 1 Department of Chemistry and Biochemistry, The University of Alabama, Tuscaloosa, AL 35487, USA; 2 Department of Chemistry and Biochemistry, The University of Texas Arlington, Arlington, TX 76019, USA; 3 Department of Chemical and Biological Engineering, The University of Alabama, Tuscaloosa, AL 35487, USA

**Keywords:** ruthenium, anticancer, light activation, protic ligands, highly conjugated ligands, luminescence

## Abstract

Protic ruthenium complexes using the dihydroxybipyridine (dhbp) ligand combined with a spectator ligand (N,N = bpy, phen, dop, Bphen) have been studied for their potential activity vs. cancer cells and their photophysical luminescent properties. These complexes vary in the extent of π expansion and the use of proximal (6,6′-dhbp) or distal (4,4′-dhbp) hydroxy groups. Eight complexes are studied herein as the acidic (OH bearing) form, [(N,N)_2_Ru(*n,n*′-dhbp)]Cl_2_, or as the doubly deprotonated (O^−^ bearing) form. Thus, the presence of these two protonation states gives 16 complexes that have been isolated and studied. Complex **7_A_**, [(dop)_2_Ru(4,4′-dhbp)]Cl_2_, has been recently synthesized and characterized spectroscopically and by X-ray crystallography. The deprotonated forms of three complexes are also reported herein for the first time. The other complexes studied have been synthesized previously. Three complexes are light-activated and exhibit photocytotoxicity. The log(D_o/w_) values of the complexes are used herein to correlate photocytotoxicity with improved cellular uptake. For Ru complexes **1**–**4** bearing the 6,6′-dhbp ligand, photoluminescence studies (all in deaerated acetonitrile) have revealed that steric strain leads to photodissociation which tends to reduce photoluminescent lifetimes and quantum yields in both protonation states. For Ru complexes **5**–**8** bearing the 4,4′-dhbp ligand, the deprotonated Ru complexes (**5_B_**–**8_B_**) have low photoluminescent lifetimes and quantum yields due to quenching that is proposed to involve the ^3^LLCT excited state and charge transfer from the [O_2_-bpy]^2−^ ligand to the N,N spectator ligand. The protonated OH bearing 4,4′-dhbp Ru complexes (**5_A_**–**8_A_**) have long luminescence lifetimes which increase with increasing π expansion on the N,N spectator ligand. The Bphen complex, **8_A_**, has the longest lifetime of the series at 3.45 μs and a photoluminescence quantum yield of 18.7%. This Ru complex also exhibits the best photocytotoxicity of the series. A long luminescence lifetime is correlated with greater singlet oxygen quantum yields because the triplet excited state is presumably long-lived enough to interact with ^3^O_2_ to yield ^1^O_2_.

## 1. Introduction

Light-activated ruthenium complexes show great promise in their ability to target cancer cells [1] via singlet oxygen generation in photodynamic therapy (PDT) [2,3,4] or via generation of toxic species via photoactivated chemotherapy (PACT) [5,6,7,8,9]. This paper focuses primarily on PDT and correlating singlet oxygen production with other photophysical properties, including photoluminescence quantum yield and the photoluminescence lifetime.

**Figure 1:**
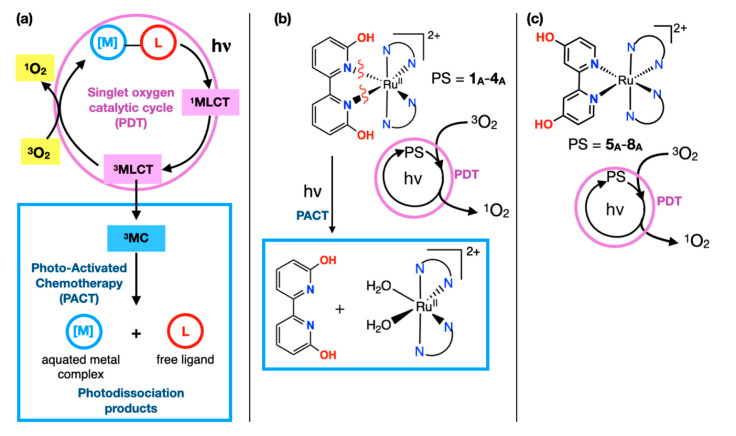
Ru complexes herein can utilize both PACT and PDT pathways. (**a**) Schematic showing the excited states typically involved in PACT and PDT. ^1^MLCT and ^3^MLCT are shown here for concise presentation, but singlet oxygen formation often occurs via ^3^ILCT and other excited states (e.g., ^3^LLCT), especially with highly conjugated organic ligands. (**b**) A specific example showing PACT and PDT for a Ru(II) complex; thus, the complex is a dual PDT/PACT agent (e.g., complexes **1_A_**–**4_A_** herein). (**c**) Complexes **5_A_**–**8_A_** used herein are solely PDT agents due to a lack of steric strain near the metal center. The co-ligands in **1_A_**–**8_A_** are defined in Figure 2.

PDT represents a catalytic cycle that does not consume the photosensitizer (PS, e.g., the Ru complex as used herein), as shown in Figure 1a. Typically, a metal complex is excited to a ^1^MLCT (metal to ligand charge transfer) state, and then intersystem crossing to the ^3^MLCT is usually fast and occurs with high efficiency for Ru tris diimine complexes [10]. The ^3^MLCT state is shown here (Figure 1a) for the sake of simplicity, but some metal complexes with extended π systems on the ligands also involve other low-lying excited states, including ^3^LLCT (ligand to ligand charge transfer) and ^3^ILCT (intra-ligand charge transfer) [11,12,13,14]. These triplet excited states can all transfer energy to ^3^O_2_ and generate ^1^O_2_ as a toxic species [15,16]. The lifetime of these triplet excited states can, in some cases, be measured by photoluminescence spectroscopy, and a longer-lived triplet excited state (in the absence of a quencher) corresponds to a greater probability of singlet oxygen formation if other non-radiative decay processes are negligible. This cycle is shown in the purple circle in Figure 1a.

Alternatively, for certain complexes, the ^3^MLCT (or other triplet excited states) can relax to a ^3^MC (metal-centered) state, as shown in the blue square in Figure 1a. This pathway typically becomes important for complexes with steric strain near the metal center [5,17,18,19]. The ^3^MC state is typically antibonding between the Ru center and the ligand with steric strain or weaker bonds [11]. This state can lead to photodissociation of the organic ligand, which produces a solvated metal complex and free ligand (Figure 1a, blue box). These species can be toxic by either generating a labile Ru center that can bind to biomolecules or by liberating toxic organic ligands [20,21,22,23,24].

**Figure 2:**
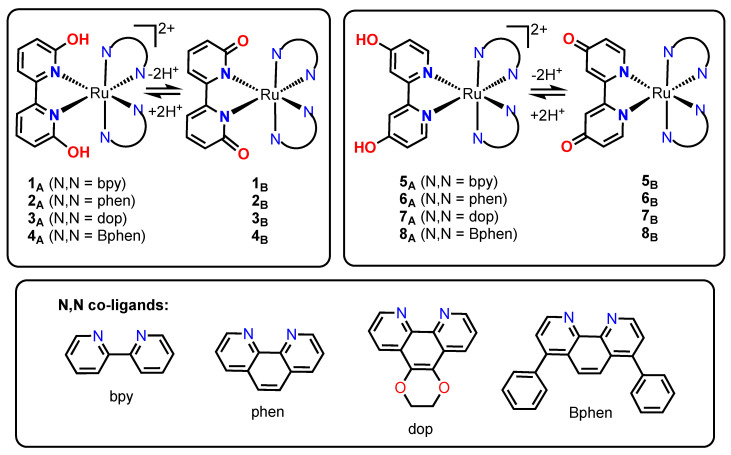
Ruthenium complexes **1**–**8** are used in the current study. Complexes **1_A_**–**4_A_**, [(6,6′-dhbp)Ru(N,N)_2_]Cl_2_, are shown in the top left, along with their basic deprotonated forms, **1_B_**–**4_B_**, which are neutral species. Complexes **5_A_**–**8_A_**, [(4,4′-dhbp)Ru(N,N)_2_]Cl_2_, are shown in the top right, along with their basic deprotonated forms, **5_B_**–**8_B_**, which are neutral species. The N,N co-ligands used herein are shown in the bottom box.

Complexes **1_A_**–**4_A_** (Figure 1b and Figure 2) utilize the 6,6′-dhbp (dhbp = dihydroxybipyridine) ligand with hydroxy groups near the metal center, whereas complexes **5_A_**–**8_A_** (Figure 1c and Figure 2) utilize the 4,4′-dhbp ligand with distal hydroxy groups [11,25,26,27,28,29]. We chose the N,N spectator ligands as bpy, phen, dop, and Bphen (as shown in Figure 2) to gradually increase π expansion to improve singlet oxygen generation and cellular uptake (vide infra) [11,26]. Both dhbp ligands are diprotic, and the use of protic Ru anticancer compounds has been rare [30]. Double deprotonation of the **A** form (e.g., **1_A_** which is dicationic, Figure 2) produces the neutral **B** form (e.g., **1_B_**). The terminology **X_A_** is used for the isolated dications (**1_A_**–**8_A_**), **X_B_** is used for the isolated neutral species (**1_B_**–**8_B_**), and a lack of subscript indicates an equilibrium mixture of **A** and **B** forms as dictated by the solution pH and the p*K*_a_ values. The measured p*K*_a_ values are given in the sixth column of Table 1, and they are typically ~5–6 [25,26,28]. This indicates that complexes **1**–**8** are predominantly doubly deprotonated at physiological pH, but there can still be a significant amount of the OH bearing **A** form and the mono-deprotonated cationic species at physiological pH [29]. Herein, luminescence studies are carried out in rigorously dried acetonitrile, and thus the isolated **A** and **B** forms should stay as such. Thus, this allows for a fundamental study of how protonation state influences the photophysics.

The presence or absence of sterically demanding groups near the metal center influences whether photodissociation occurs. The 6,6′-dhbp (dhbp = dihydroxybipyridine) ligand in **1_A_**–**4_A_** induces steric strain near the metal center. These complexes photodissociate (blue box in Figure 1b) but the quantum yields (Φ_PS_) are low (Table 1) [28]. These complexes produce higher quantum yields of singlet oxygen (Φ_Δ_) (purple circle in Figure 1b and Table 1) vs. photodissociation (Φ_PS_), and singlet oxygen generation appears to be responsible for the observed photocytotoxicity (only **3_A_** and **4_A_** show significant photocytotoxicity based on PI values, PI = phototoxicity index = EC_50_dark_/EC_50_light_, Table 1 and vide infra) [11,26]. This difference in toxicity across this series is proposed to be due primarily to uptake. It has been measured in our past work and estimated by log(D_o/w_) values (Table 1) [29]. Complexes **3_A_** and **4_A_** are the most lipophilic of the **1_A_**–**4_A_** series, and lipophilic compounds are predicted to pass most readily through the phospholipid cell membrane by passive diffusion. An ideal range for log(D_o/w_) is typically given as 2–6, so long as they have sufficient water solubility for drug delivery [31,32,33,34]. The distribution coefficient (D_o/w_) is used for ionizable metal complexes, whereas P (partition coefficient) is used for aprotic, nonionizable metal complexes.

Complexes **5_A_**–**8_A_** lack steric strain near the metal center and, therefore, do not undergo photodissociation under the conditions used herein [25,26,28,35]. Thus, Figure 1c shows only the singlet oxygen generation pathway for these compounds. While the protonation state of complexes (**1_A_**–**8_A_** vs. **1_B_**–**8_B_** in Figure 2 and Table 1) has an influence on these pathways, this has been described in our past work and further discussion is best deferred to the main text. In our past work, the reasons for the lack of toxicity observed for complexes **5_A_** and **6_A_** were elusive. Herein, we report log(D_o/w_) values and singlet oxygen quantum yields (Φ_Δ_) to better explain these trends. Likewise, complex **7_A_** is new, and here we report its synthesis, characterization data, log(D_o/w_) values, singlet oxygen quantum yields (Φ_Δ_), and photocytotoxicity data. Complexes **5_A_**–**7_A_** are contrasted with **8_A_**, which is our most photocytotoxic compound (PI > 200). This work aims to explain these trends in terms of log(D_o/w_) values to estimate cellular uptake. Furthermore, for all sixteen complexes (**1_A_**–**8_A_**, **1_B_**–**8_B_**), we have performed photoluminescence studies to understand the photophysics and to correlate photoluminescence parameters with singlet oxygen quantum yields.

## 2. Results and Discussion

### 2.1. Synthesis

The protic 6,6′-dhbp ligand was combined with four spectator ligands (N,N = bpy, phen, dop, BPhen) to produce [(N,N)_2_Ru(6,6′-dhbp)]Cl_2_ complexes (**1_A_**, **2_A_**, **3_A_**, and **4_A_**, respectively) using previously reported synthesis procedures (see Figure 2 for structures) [25,26,28]. These complexes with OH groups near the metal center are contrasted herein with the 4,4′-dhbp complexes: [(N,N)_2_Ru(4,4′-dhbp)]Cl_2_ complexes (**5_A_**, **6_A_**, **7_A_**, and **8_A_**, using N,N = bpy, phen, dop, and BPhen, respectively, Figure 2). Complexes **5_A_**, **6_A_** and **8_A_** were reported previously [25,26,28], whereas complex **7_A_** is new [35]. Complex **7_A_** was prepared by refluxing (dop)_2_RuCl_2_ (1.0 equiv.) with 4,4′-dhbp (1.2 equiv.) in either water or 50/50 ethanol/H_2_O. Following purification by filtration to remove unreacted 4,4′-dhbp and washing with water, the dark orange-red solid was recrystallized by vapor diffusion of diethyl ether into a saturated ethanol solution to produce dark red X-ray quality crystals (60% yield). Single crystal X-ray diffraction data are presented below.

The deprotonated basic forms of these complexes (see Table 1 for p*K*_a_ values) were prepared and isolated as described previously for **1_B_**–**4_B_** and **8_B_** [11,26]. Typically, the acidic forms of the complexes are treated with NaOH_(*aq*)_ in methanol or ethanol to generate the basic forms which are then isolated and purified. A similar procedure was employed for **5_A_**–**7_A_** which were treated with NaOH_(*aq*)_ with the pH adjusted to 8.2. A dark purple suspension resulted which was filtered (to remove NaCl_(*aq*)_ as the byproduct in aqueous solution) and then the dark purple solid was washed with diethyl ether and hexanes. After drying under vacuum, complexes **5_B_**–**7_B_** were isolated and characterized. These samples were used to measure singlet oxygen quantum yields (vide infra).

### 2.2. Characterization of the Compounds

The purity and identity of the new (**5_B_**–**7_B_** and **7_A_**) and previously reported (**1_A_**–**6_A_**, **8_A_**, **1_B_**–**4_B_**, and **8_B_**) samples were confirmed using ^1^H NMR, IR, UV-Vis spectroscopy and HRMS. Spectra for the new complexes are shown in the Supporting Information.

### 2.3. Crystallography

**Figure 3:**
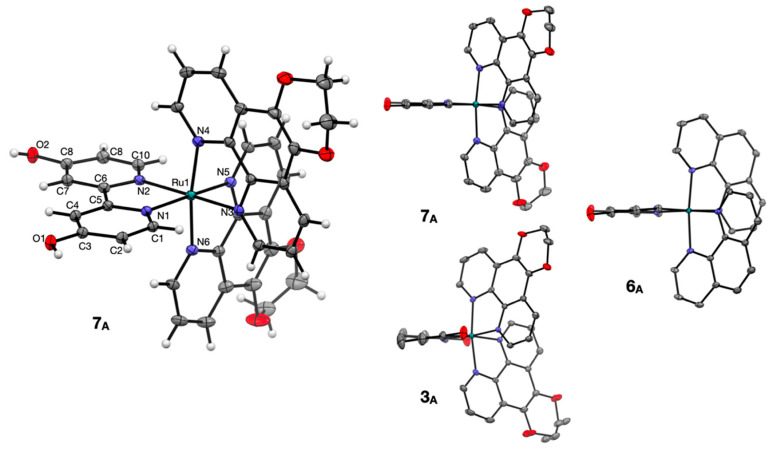
Molecular diagrams for **7_A_** crystallized as [(dop)_2_Ru(4,4′-dhbp)]Cl_2_(H_2_O)_3_. In both views of **7_A_**, water solvent and chloride counter anions have been removed for clarity. In the right-hand view, hydrogen atoms are hidden for clarity. Ellipsoids are shown at 50% probability. Molecular diagrams of **3_A_** and **6_A_** (from prior publication [28]) are included for comparison. Grey = carbon, white = hydrogen, red = oxygen, blue = nitrogen, teal = ruthenium.

Complex **7_A_** was recrystallized as dark red blocks suitable for single crystal X-ray diffraction (SC XRD) by vapor diffusion of diethyl ether into a saturated ethanol solution of **7_A_**. The molecular diagram for complex **7_A_** is shown in Figure 3 and is compared with complexes **3_A_** and **6_A_** which were previously published [28]. The molecular packing diagram shows that the complex **7_A_** has OH to Cl^-^ hydrogen bonds (O1 to Cl1 = 2.959(2) Å and O2 to Cl1 = 3.008(2) Å) as well as Cl^−^ to H_2_O (solvate) hydrogen bonds. Thus, each chloride ion has three hydrogen bonding interactions in total. The interactions between OH (of 4,4′-dhbp) and chloride are indicative of a strong hydrogen bonding interaction [28,36,37].

The ruthenium center in **7_A_** displays a distorted octahedral geometry as shown by the bond lengths and angles in Table 2. Slight distortions away from ideal 90° bond angles are due to chelation which leads to N-Ru-N angles of 78–80° within the chelate rings. Outside of the chelate rings, the N-Ru-N *cis* angles are ~89–98° and the *trans* N-Ru-N angles are 172–174°. The Ru-N bond distances (~2.05–2.07 Å) and the above angles are quite similar to the closely related compound **6_A_** [28]. The C-O distances (Table 2) indicate that π donation via resonance from the pyridinol oxygen to carbon results in a partial C=O double bond character (C-O = ~1.34 Å lies between a single and double bond, see Table 2). This C-O distance is quite similar to other pyridinol containing compounds [25,26,28,36,38,39]. When both **6_A_** and **7_A_** are viewed side on with the 4,4′-dhbp ligand lined up (Figure 3, right hand side) it is clear that they both show relatively little twisting of the 4,4′-dhbp ligand, in contrast to the 6,6′-dhbp ligand in **3_A_** which is twisted such that both pyridinol rings are not coplanar [28]. Furthermore, the steric congestion in **3_A_** results in N donor groups of the dop ligand being forced away from ideal octahedral symmetry. Since such steric distortion correlates with photodissociation by lowering the energy of the dissociative ^3^MC state, it is not surprising that **3_A_** photodissociates but **6_A_** and **7_A_** (and **5_A_** and **8_A_**, which lack crystal structures) are photostable (as shown in Figure 1) [25,26,28]. Our past work has described photodissociation of **3_A_** involving visible light (λ_max_ = 450 nm) triggered dissociation of 6,6′-dhbp to yield an aqua complex (Figure 1a,b).

### 2.4. UV-Vis Spectroscopy

The UV-Vis spectrum of each diprotic compound (**1_A_**–**8_A_**) in acetonitrile displays a singlet metal-to-ligand charge transfer (^1^MLCT) band (λ_abs_ = 455–483 nm) (Table S1, Figures S7–S9). A bathochromic shift and peak broadening of the absorption band was observed for the doubly deprotonated forms (**1_B_**–**8_B_**) (Table S1, Figures S7–S9). This shift is attributed to the population of a ^1^LLCT band (λ_abs_ = 515–548 nm) as confirmed computationally for compounds **2_B,_ 3_B_**, **4_B_** and **8_B_** [11,26]. The ^1^LLCT results from electron transfer from a molecular orbital (MO) centered on the deprotonated *n,n*′-dhbp (n = 4 or 6) to the N,N spectator ligand.

### 2.5. Singlet Oxygen Quantum Yields

Singlet oxygen quantum yields were reported previously for compounds **1**–**4** and **8** (Table 1) [11,26]. With the 6,6′-dhbp co-ligand, these values increase with increasing π expansion from **1** (bpy coligand) to **2** (phen) and **3** (dop) (Figure 2, Table 1) [11]. Compounds **1**–**3** were measured in CD_3_OD and are, therefore, not directly comparable with compounds **4**–**8** measured in CH_3_CN. In our continuing work, CH_3_CN is preferred due to a lack of H/D exchange or protonation/deprotonation events. Comparing **4** and **8** (Bphen and either 6,6′-dhbp or 4,4′-dhbp, respectively, as coligands), we observed that the Φ_Δ_ values are higher for **8** (Φ_Δ_ = 68% for **8_A_**) vs. **4** (Φ_Δ_ = 5% for **4_A_**) due to moving the OH groups to the periphery which relieves steric congestion near the metal and prevents photodissociation of a ligand [26]. With the 4,4′-dhbp co-ligand, compounds **5**–**7** were measured for this work, and show that the Φ_Δ_ values again increase with π expansion from **5_A_** (bpy coligand, Φ_Δ_ = 57%) to **6_A_** (phen, Φ_Δ_ = 62%) and finally to **7_A_** (dop, Φ_Δ_ = 76%) (Figure 2, Table 1). For compounds **5**–**8**, reduced Φ_Δ_ values are observed for the **B** forms relative to the **A** forms in Table 1. These values are discussed further below in the context of photoluminescence and light-induced cytotoxicity trends.

### 2.6. Photoluminescence, Photoluminescence Quantum Yield, and Luminescence Lifetime Studies

**Figure 4:**
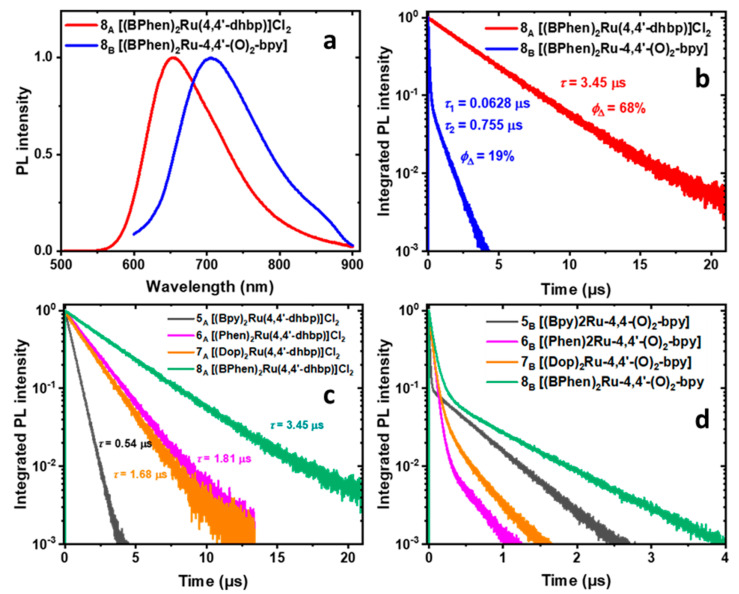
Rationalizing the trends in Φ_Δ_ of compounds **5_A_**–**8_A_** in terms of their photoluminescence (PL) dynamics. (**a**) Steady-state PL spectra of **8_A_** and **8_B_** in deaerated acetonitrile excited at their corresponding absorption peak wavelengths (483 nm and 525 nm, respectively). (**b**) Comparison of the time-resolved PL dynamics of **8_A_** and **8_B_** excited at 404 nm. (**c**) Time-resolved PL dynamics of **5_A_**–**8_A_** excited at 404 nm. (**d**) Time-resolved PL dynamics of **5_B_**–**8_B_** excited at 404 nm.

**Figure 5:**
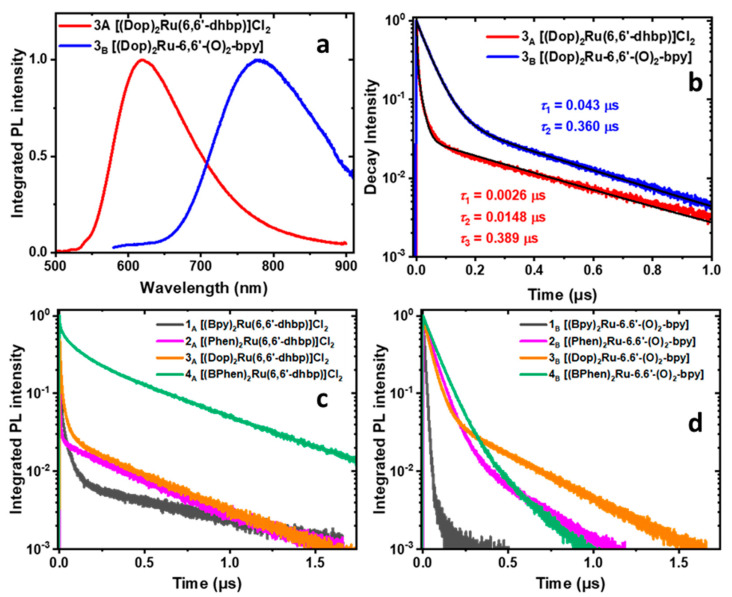
(**a**) Steady-state PL spectra of **3_A_** and **3_B_** excited at at their corresponding absorption peak wavelengths (467 nm and 529 nm, respectively). (**b**) Comparison of the time-resolved PL dynamics of **3_A_** and **3_B_** excited at 404 nm. (**c**) Time-resolved PL dynamics of **1_A_**–**4_A_** excited at 404 nm. (**d**) Time-resolved PL dynamics of **1_B_**–**4_B_** excited at 404 nm.

To gain insights into the observed variation of Φ_Δ_, we measured the steady-state and time-resolved PL of all 16 compounds (Table S1). The PL spectra of compounds **1_A_**–**8_A_** in acetonitrile displayed a band centered at 610–650 nm (e.g., Figure 4a and Figure 5a, Figures S10–S13) due to ^3^MLCT emission. Doubly deprotonated forms **5_B_–8_B_** demonstrated a red shift (*λ*_em_ = 704–775 nm) and broadening of their PL bands compared with the protonated forms (Figure 4a and Figure 5a, Figures S10–S13). This red shift is proposed to be due to the generation of the triplet ligand-to-ligand charge transfer excited state (^3^LLCT) by the mixing of the *n*,*n*′-dhbp (n = 4 or 6) with the N,N ligands (bpy, phen, dop, or BPhen), as suggested by computations on the excited states of **2_,_ 3**, **4**, and **8** [11,26]. The electron donation from the deprotonated *n*,*n*′-dhbp (namely [O_2_-bpy]^2−^) to the ancillary (N,N) ligands leads to the generation of an electron density capable of quenching the emission of the doubly deprotonated compounds by a photoinduced electron transfer mechanism (PET) followed by nonradiative decay via back electron transfer [12,40].

The PLQY (Φ_PL_) of compounds **5_A_**–**8_A_** ranged from 5.3% to 18.7% (Table 3). The Φ_PL_ of compounds **5_A_**–**8_A_** increases with greater π-expansion of the ancillary ligands in the order **5_A_** (bpy) < **6_A_** (phen) ≅ **7_A_** (dop) < **8_A_** (BPhen). These values, when compared with values near zero for compounds **1_A_**–**4_A_** (see Table S1), show that π-expansion coupled with a lack of Ru-N strain is essential for improved Φ_PL_. In time-resolved PL measurements, these compounds (**5_A_**–**8_A_**) were unique as they showed monoexponential PL dynamics, with lifetimes ranging from 0.538 to 3.45 μs (Figure 4b,c, Figures S22, S24, S26, S28, Table S1). A monoexponential PL decay suggests a homogeneous ensemble of emitters with parallel radiative and non-radiative (decay to the non-emissive ^3^MC) processes. The radiative recombination rate of compounds **5_A_**–**8_A_** can be calculated as *τ*_rad_ = *τ*_meas_/Φ_PL_. The calculated radiative lifetimes are more coherent, ranging from 10.2 to 18.5 μs (Table 3), which is comparable with the radiative lifetime of [(bpy)_3_Ru]Cl_2_ (9.4 μs). The long radiative lifetimes are in accord with the high singlet-oxygen quantum yields (Φ_Δ_ = 57–76%) of **5_A_**–**8_A_**: the slow radiative recombination cannot compete with energy transfer from ^3^MLCT to ^3^O_2_ in these compounds.

In contrast to **5_A_**–**8_A_**, deprotonated compounds **5_B_**–**8_B_** show a biexponential PL decay with the fast component in the range from 0.010 µs to 0.063 µs (Figure 4b,d, Figures S23, S25, S27, S29, see Table S1). The PLQY (Φ_PL_) of these compounds is lower than 1%, which suggests that the fast component in the PL decay is dominated by a non-radiative process. This observation is in accord with the lower singlet-oxygen QYs measured for these compounds: the fast sub-0.1 µs depopulation of the ^3^MLCT state competes with energy transfer to ^3^O_2_. The biexponential PL dynamics indicate the presence of two emission channels. In the literature, it has been attributed to the presence of the low-emitting ^3^LLCT state [5,11,26]. Our observed fast times of the order of 10^1^ ns are too long to be ^3^MLCT to ^3^LLCT transfer (30–200 ps) [12]. The biexponential decay likely comes from depopulation of the ^3^MLCT and ^3^LLCT states produced independently of one another in different photoexcitation events. The PL spectra of deprotonated compounds **5_B_**–**8_B_** show a weak peak on their red shoulder (e.g., at 850 nm for **5_B_**), which could have different PL dynamics. To examine the contribution from this peak, we collected the PL dynamics with a 715 nm long-pass filter but did not see a difference in the lifetimes or their amplitudes from the PL dynamics collected with a 645 nm long-pass filter. This suggests that the collected PL dynamics are uniform over the entire PL spectrum.

In contrast to **5_A_**–**8_A_**, compounds **1_A_**–**4_A_** show a biexponential PL decay with the fast component in the single-nanosecond range (Figure 5b,c, Figures S14, S16, S18, S20, Table S1). The PLQY of these compounds is lower than 1%, which suggests that the fast component in the PL decay is dominated by a non-radiative process, likely an intersystem crossing from ^3^MLCT to the non-emissive ^3^MC state [11,41]. Again, this observation is in accord with the lower singlet-oxygen QYs (Φ_Δ_) measured for these compounds: the fast ns-scale depopulation of the ^3^MLCT state competes with energy transfer to ^3^O_2_. Interestingly, unlike compounds **4–8** the fast PL decay component of the basic forms **1_B_**–**3_B_** ranges from 11 to 57 ns, which is an order of magnitude longer than the fast component of the acidic forms **1_A_**–**3_A_** (Figure 5c,d, Figures S15, S17, S19, Table S1). This observation agrees with the measured order-of-magnitude higher singlet-oxygen QYs of compounds **1_B_**–**3_B_** (measured in CD_3_OD, Table 1) [11].

For all compounds, we observed a strong correlation between the singlet-oxygen QY (Φ_Δ_) and their PL dynamics: compounds with high Φ_Δ_ do not show fast (sub-10 ns) components in their PL dynamics. Can we quantify this observation? The Φ_Δ_ measured in aerated solvent is the ratio of the ^3^MLCT to ^3^O_2_ transfer rate (*γ*_O2_) to the sum of all ^3^MLCT depopulation rates which includes γ_O2_ and depopulation by other means (*γ*_other_) (Equation (1)):

(1) $$\Phi_{\Delta }=\frac{\gamma_{O2}}{\gamma_{O2}+\gamma_{other}}$$

The depopulation rate of ^3^MLCT through channels other than energy transfer to ^3^O_2_ is determined from the fast component of the PL dynamics in deaerated solvent. Taking the reciprocal of both sides, we obtain Equation (2), where τ_O2_ = 1/γ_O2_ and τ_1_ = 1/γ_other_ (τ_1_ is the lifetime for the fast component which best captures the decay due to processes other than transfer to ^3^O_2_):

(2) $$1/\Phi_{\Delta }=1+\tau_{O2}/\tau_{1}$$

**Figure 6:**
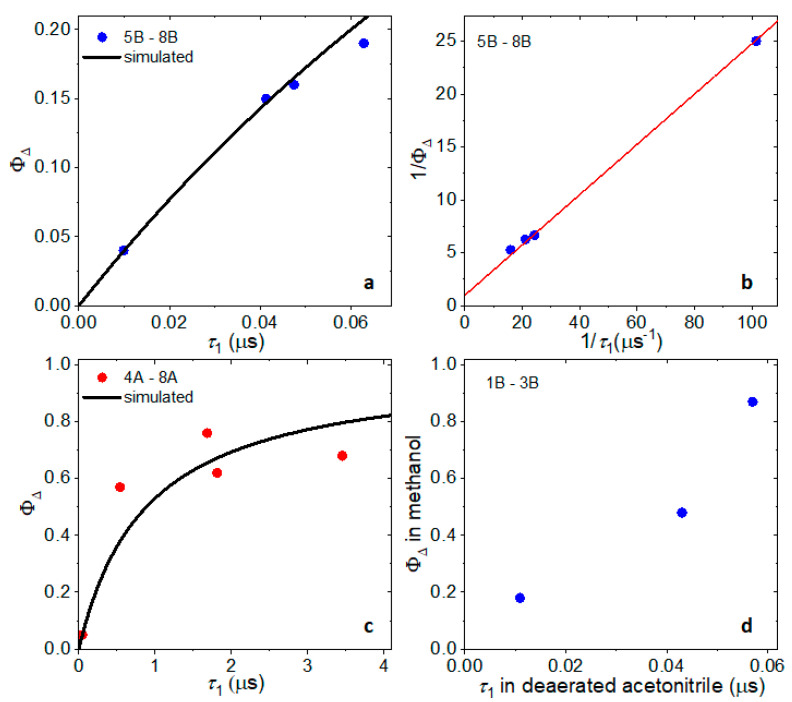
Correlation between the PL lifetime and singlet-oxygen QY (Φ_Δ_). (**a**) Φ_Δ_ of compounds **5_B_–8_B_** in aerated acetonitrile as a function the fast PL dynamics lifetime of these compounds in deaerated acetonitrile. The line shows calculated ϕ_Δ_ assuming the transfer time from ^3^MLCT to ^3^O_2_ equals 0.240 µs (from the analysis in (**b**)). (**b**) Data in (**a**) plotted on the reciprocal scales to determine the “average” ^3^MLCT to ^3^O_2_ transfer time. (**c**) Φ_Δ_ of compounds **4_A_**–**8_A_** in aerated acetonitrile as a function the fast PL dynamics lifetime of these compounds in deaerated acetonitrile. The line shows calculated Φ_Δ_ assuming the transfer time from ^3^MLCT to ^3^O_2_ equals 0.884 µs. (**d**) Φ_Δ_ of compounds **1_B_**–**3_B_** in aerated methanol (CD_3_OD) as a function the fast PL dynamics lifetime of these compounds in deaerated acetonitrile.

The energy transfer time to ^3^O_2_ is different for all compounds and depends on the concentration of dissolved O_2_, so it cannot be determined from a dataset for different compounds. In Figure 6b, we estimate the “average” ^3^MLCT to ^3^O_2_ transfer time under the measurement conditions to be 0.240 µs for compounds **5_B_**–**8_B_** (τ_O2_ is estimated from the slope in Figure 6b as follows from Equation (2)). Similarly, the “average” ^3^MLCT to ^3^O_2_ transfer time equals 0.884 µs in compounds **5_A_**–**8_A_**. Using these estimated values, we can estimate the ^3^O_2_ QY from the fast component in the PL dynamics (solids lines in Figure 6a,c). Finally, even though ^3^O_2_ QY of **1_B_**–**3_B_** was measured in methanol and PL dynamics in acetonitrile, we observe the same trend: the slower the fast component in the PL decay, the higher the ^3^O_2_ QY in compounds **1_B_**–**3_B_** (Figure 6d).

### 2.7. Cellular Cytotoxicity and Photocytotoxicity Data as Related to the Log(D_o/w_) Values and Photophysical Properties of These Compounds

The ruthenium complexes were studied as potential anticancer agents in breast (MCF7) and cervical cancer (HeLa) cell lines (Table 1). For all the ruthenium complexes except **7**, the cell studies and mode of action studies were reported in previous papers [5,11,25,26,27,28]. Selected data are shown in Table 1, including the cell line studied, the EC_50_light_ (studied with white or blue light, see Table 1 footnotes), the EC_50_dark_, and the phototoxicity index (PI = EC_50_dark_/EC_50_light_). These values for complex **7** are also reported herein in recent studies [35]. All these complexes **1**–**8** are given to cells as the acidic form, **1_A_**–**8_A_**, but in cellular media they deprotonate and form a mixture of different protonation states consisting of mostly the deprotonated forms (**1_B_**–**8_B_**) at pH 7.4–7.5. The average p*K*_a_ values are typically 5.9–6.3, as reported previously and shown in the sixth column of Table 1 [25,26,28].

With the 6,6′-dhbp ligand, compounds **1** and **2** are effectively non-toxic both in the dark and upon light irradiation. While these compounds are not very effective at photosubstitution (Φ_PS_ is 10^−3^ to 10^−1^% as shown in the eighth column of Table 1), they do produce singlet oxygen to a significant extent (Φ_Δ_ as high as 87% for **2_B_**) [11,25,28]. Thus, their lack of toxicity was attributed to low uptake as measured by ICP-MS and flow cytometry and consistent with the lower log(D_o/w_) values of 1.4 and 1.6 at pH 7.4 [11,28,29]. Compounds **3** and **4** both exhibit light activated toxicity with PI values of 120 and 9 vs. MCF-7 breast cancer cells [26,28]. Both compounds have comparable EC_50_light_ values of 2–4 μM, but **4** has greater dark toxicity (via an unknown mode of action), which reduces the PI value. For compound **3**, the toxicity is primarily attributed to singlet oxygen formation (Φ_Δ_ = 48% for **3_B_**), whereas the cause of toxicity for **4** is less clear but may include singlet oxygen formation (Φ_Δ_ = 5% for **4_A,_** <1% for **4_B_**) [11,26]. The Φ_Δ_ values are not directly comparable for **1**–**3** vs. **4** because of a difference in solvent used, with quantum yields for **1**–**3** measured in CD_3_OD and **4** measured in CH_3_CN due to solubility constraints. Overall, the improved PI values of **3** and **4** (relative to **1** and **2**) are attributed to improved uptake (as measured by log(D_o/w_) of 1.8 and >3 for **3** and **4**, respectively) and localization in certain organelles. Fluorescence microscopy studies have shown that **3** localizes in the nucleus and **4** localizes in the mitochondria of cells [26,28,29].

Examining the Ru complexes with the 4,4′-dhbp ligand (**5**–**8**), we can determine the impact of moving the OH groups to the periphery. The values of log(D_o/w_) and Φ_Δ_ are reported in Table 1 for the first time for **5**–**7**, and furthermore, all synthesis, characterization, and toxicity data for **7** are new. Complexes **5**–**7** are all effectively non-toxic in the dark and upon light irradiation with PI values near 1 [25,28,35]. This is despite efficient singlet oxygen production in acetonitrile for both the A and B forms (**5_A_**–**7_A_** have Φ_Δ_ ranging from 57–76% and **5_B_**–**7_B_** have Φ_Δ_ ranging from 4–19%). Moving the OH groups to the 4-position on the pyridinol rings results in negligible photodissociation under irradiation with visible light using the same light dosage as for our cell studies (footnotes b and c in Table 1). Thus, for **5**–**7**, the lack of toxicity can be attributed to poor uptake as is consistent with log(D_o/w_) values of 0.8 and lower. These are relatively hydrophilic compounds with poor uptake predicted. It appears that moving the OH groups to the periphery (more solvent accessible) has the impact of reducing the log(D_o/w_) value by 1–2 units (cf. **5** vs. **1**, **6** vs. **2**, **7** vs. **3**, in Table 1, seventh column). A difference is solvent precludes a comparison between Φ_Δ_ values for **1**–**3** vs. **5**–**7**. However, CH_3_CN was used as the solvent for **4** and **8**, and we can see that moving OH group to the periphery improved Φ_Δ_ values by 14-fold from **4_A_** to **8_A_** by removing the photodissociation pathway, as shown in Figure 1.

Complex **8** represents the first complex of 4,4′-dhbp to have good light activated toxicity and a PI value of >200 vs. MCF-7. In this compound, effective singlet oxygen formation (Φ_Δ_ = 68% for **8_A_**) is combined with good uptake (log(D_o/w_) > 3) and localization in the mitochondria of the cells, as shown by fluorescence microscopy [26]. Each factor can be thought of as necessary but not sufficient for toxicity. Singlet oxygen production is necessary, but complexes **5**–**7** lack favorable uptake, and typically, singlet oxygen production outside the cell does not lead to toxicity.

## 3. Materials and Methods

### 3.1. Synthesis and Characterization Data

#### 3.1.1. General

The chemicals 2,2′-Bipyridine (bpy), 1,10-phenanthroline (phen), and magnesium sulfate were purchased from Sigma-Aldrich (St. Louis, MI, USA). Bathophenanthroline and 6,6-dihydroxy-2,2′-bipyridine were purchased from Tokyo Chemical Industry (Tokyo, Japan), Ruthenium trichloride hydrate was purchased from Pressure chemical (Pittsburgh, PA, USA), 4,4′-dihydroxy-2,2′-bipyridine was purchased from Combi-Blocks (San Diego, CA, USA). All ligands and metal sources purchased from commercial sources were used as received. The dop ligand was synthesized as previously reported [42]. Sodium hydroxide pellets were purchased from VWR Chemicals (Solon, OH, USA). Solvents were purchased from VWR. NMR spectra were recorded on a Bruker AVANCE 360 (360 MHz, ^1^H frequency) or AVANCE 500 (500 MHz, ^1^H frequency) (Bruker is in Billerica, MA, USA). UV-Vis spectra were recorded on a Jasco V-780 spectrophotometer (Jasco Corporation, Tokyo, Japan) using a quartz cuvette (Venier Software, ON, USA) of 1 cm path length under ambient atmosphere. Infrared spectra were taken using a Bruker Alpha FT-IR spectrometer (Germany). Compounds **1_A_**, **1_B_**, **2_A_**, **2_B_**, **3_B_**, **4_A_**, **4_B_**, **5_A_**, **6_A_**, **8_A_**, and **8_B_** have been synthesized and characterized as reported previously [11,25,26,28]. Thus, here we focus on the synthesis and isolation of compounds **5_B_**, **6_B_**, **7_A_**, and **7_B_**.

#### 3.1.2. Synthesis and Characterization of 7_A_ = [(dop)_2_Ru(4,4′-dhbp)]Cl_2_

The starting material (dop)_2_RuCl_2_ was prepared as previously reported [28,43]. The complex (dop)_2_RuCl_2_ (0.0949 g, 0.1458 mmol) was treated with 1.2 mole equivalent 4,4′-dhbp (0.0329 g, 0.1749 mmol) and 12 mL of degassed deionized water under air-free conditions. Light was excluded as a precaution, although later studies have shown that the products of this reaction are light-stable. The dark purple solution was refluxed at 110 °C for 18 h. A drop of 5M HCl was added to the red solution after the reaction was cooled to room temperature. The solution was filtered to remove excess ligand as a solid, washed with cold water, and the water from the filtrate was removed by evaporation to give a red solid. This red solid was then washed with hexane and diethyl ether and dried in vacuum to produce 0.0264 g (0.0315 mmol) of **7_A_** at 22% yield. The product was recrystallized for further purification. The above procedure was used for all photoluminescence experiments and other spectroscopy studies on **7_A_**. The sample of **7_A_** used for single crystal X-ray diffraction was prepared similarly, but the reaction was run in 1:1 EtOH to water to give a 60% yield. Dark red X-ray quality crystals were grown by vapor diffusion of Et_2_O into a saturated ethanol solution.

^1^H-NMR on **7_A_** (360 MHz, DMSO-d_6_) δ: 12.16 (s, 2H), 8.69 (d, 2H, 8.4 Hz), 8.57 (d, 2H, 5.5 Hz), 8.22 (d, 2H, 5.2 Hz), 8.08 (d, 2H, 2.7 Hz), 7.92 (q, 2H, 3.2 Hz, 5.2 Hz), 7.83 (d, 2H, 5.2 Hz), 7.64 (q, 2H, 13.7 Hz, 5.3 Hz, 3.1 Hz, 5.3 Hz), 7.24 (d, 2H, 6.4 Hz), 6.89 (dd, 2H, 6.3 Hz, 2.5 Hz, 2.7 HZ), 4.69 (s, 8H). MALDI-ToF MS (low res): [C_38_H_26_N_6_O_6_RuH]^+^ *m/z* = 765.3 (Calculated *m/z* = 765.1). High res. MS: 765.1031 (calculated: 765.1036) corresponds to C_38_H_27_N_6_O_6_Ru [M-H]^+^. IR (ν, cm^−1^): 3069, 2643, 1617, 1570, 1486, 1464, 1434. Spectra are shown in the supporting information (Figures S4–S6).

#### 3.1.3. Synthesis and Characterization of the Deprotonated Complexes **5_B_**, **6_B_**, and **7_B_**

In a typical procedure, 10 mg of **5_A_**, **6_A_**, or **7_A_** was treated with ~15 mL of distilled, deionized water which was adjusted to pH 8.2 with concentrated NaOH_(*aq*)_. A dark purple suspension resulted which was filtered to remove the aqueous solution. The dark purple solid was rinsed with diethyl ether and hexanes. The solid was dried under vacuum for 6 h. Before use in luminescence experiments or for measuring singlet oxygen quantum yields, the solid was dissolved in dry acetonitrile, filtered, and the solution was dried with MgSO_4_. The solvent was removed, and the solid (**5_B_**, **6_B_**, or **7_B_**) was left under vacuum for a few hours. The identity of these compounds was confirmed by ^1^H NMR (Figures S1–S3) and IR.

^1^H-NMR on **5_B_** (360 MHz, DMSO-d_6_) δ: 8.75 (d, 2H, 5.8 Hz), 8.72 (d, 2H, 5.9 Hz), 8.06 (t, 2H, 5.6 Hz), 8.03 (d, 2H, 4.0 Hz), 7.99 (d, 2H, 4.0 Hz), 7.68 (d, 2H, 4.0 Hz), 7.65 (t, 2H, 4.8 Hz, 9.5 Hz), 7.40 (t, 2H, 4.8 Hz, 9.4 Hz), 6.61 (d, 2H, 1.9 Hz), 6.31 (d, 2H, 4.9 Hz), 5.79 (dd, 2H, 1.9 Hz, 2.9 Hz, 6.8 Hz)

^1^H-NMR on **6_B_** (360 MHz, DMSO-d_6_) δ: 8.72 (d, 2H, 5.9 Hz), 8.57 (d, 2H, 6.0 Hz), 8.46 (d, 2H, 3.8 Hz), 8.34 (d, 2H, 6.4 Hz), 8.28 (d, 6.4 Hz), 8.06 (q, 2H, 2.1 Hz, 3.8 Hz), 7.93 (d, 2H, 3.8 Hz), 7.60 (q, 2H, 2.1 Hz, 3.8 Hz), 6.70 (d, 2H, 1.8 Hz), 6.33 (d, 2H, 4.9 Hz), 5.76 (dd, 2H, 1.8 Hz, 4.8 Hz).

^1^H-NMR on **7_B_** (360 MHz, DMSO-d_6_) δ: 8.59 (d, 2H, 6.1 Hz), 8.45 (d, 2H, 5.9 Hz), 8.33 (d, 2H, 3.7 Hz), 7.99 (q, 2H, 2.2 Hz, 3.9 Hz), 7.56 (d, 2H, 3.9 Hz), 6.66 (d, 2H, 1.9 Hz), 6.21 (d, 2H, 4.9 Hz), 5.73 (dd, 2H, 1.8 Hz, 1.9 Hz, 5.1 Hz). 4.66 (s, 8H).

### 3.2. Single Crystal X-ray Diffraction

Single dark red block-shaped crystals of **7_A_** were obtained by vapor diffusion of Et_2_O into a saturated ethanol solution. A suitable crystal 0.15 × 0.10 × 0.07 mm^3^ was selected and mounted on a suitable support on an XtaLAB Synergy R, DW system, HyPix diffractometer. The crystal was kept at a steady *T* = 100.01(10) K during data collection. The structure was solved with the ShelXT [44] structure solution program using the Intrinsic Phasing solution method and by using Olex2 [45] as the graphical interface. The model was refined with version 2018/3 of ShelXL [44,46] using Least Squares minimization. All non-hydrogen atoms were refined anisotropically. H atoms were positioned geometrically and constrained to ride on their parent atoms.

The diffraction pattern was indexed, and the unit cell was refined on 27050 reflections, 45% of the observed reflections. Data reduction, scaling and absorption corrections were performed. The final completeness is 99.90% out to 30.508° in *Θ*. A gaussian absorption correction was performed using CrysAlisPro 1.171.40.49a (Rigaku Oxford Diffraction, 2019). The numerical absorption correction was based on gaussian integration over a multifaceted crystal model. Empirical absorption correction was performed using spherical harmonics as implemented in SCALE3 ABSPACK. The absorption coefficient *μ* of this material is 0.634 mm^−1^ at this wavelength (*λ* = 0.711 Å), and the minimum and maximum transmissions are 0.936 and 1.000, respectively Further crystal data are in the supporting information (Table S2).

CCDC deposition number: 2238882.

### 3.3. Spectroscopy

#### 3.3.1. Photoluminescence Measurements

The photoluminescence (PL) of the compounds was measured using a Horiba Fluoromax+ spectrofluorometer (Horiba Scientific, Glasgow, UK).

#### 3.3.2. Photoluminescence Quantum Yield Measurement

The compounds’ photoluminescence quantum yield (PLQY, Φ_PL_) was measured using a Horiba Fluoromax+ spectrofluorometer. The sample solutions were prepared in the glovebox (MBraun Unilab, Stratham, NH, USA) using acetonitrile dispensed from the solvent purification system (SPS) (Pure Process Technology, Nashua, NH, USA). About 2.5 mL of the solution was dispensed into a fluorescence cuvette equipped with a septum (Starna Cells Inc. Atascadero, CA, USA). The cuvettes were wrapped with parafilm to prevent air and moisture contamination, after which the absorbance and absorption wavelength were measured. The sample solutions were prepared such that the maximum absorbance of the samples was below 0.4 to avoid reabsorption of the PL. The PLQY measurements were performed using the comparative method. The relative process compared the absorbance and emission of the standard, [(Bpy)_3_Ru]Cl_2_, (Φ_PL_ = 9.4% in deaerated acetonitrile) with that of the sample under the same irradiance condition and was calculated using Equation (3) [47]. Equation (1) calculates the photoluminescence quantum yield of the unknown as a product of the luminescence quantum yield of the standard (Φ_s_) and the integrated emission areas (*I*), the fractions of absorbed light (*F*), and the refractive indices of the solvents (*n*) of the unknown (*X*) and the standard [48]. Here, *n_x_*/*n_s_* = 1 since the standard and the sample were measured in acetonitrile. The PLQY values are generally reproducible with a 5% error.

(3) $$\begin{array}{c} \Phi_{X}=\Phi_{S}\times \left(\frac{I_{X}}{I_{S}}\right)\times \left(\frac{F_{S}}{F_{X}}\right)\times {\left(\frac{n_{X}}{n_{S}}\right)}^{2} \end{array}$$

#### 3.3.3. Luminescence Lifetime Measurements

Luminescence lifetime measurements were carried out on a Horiba DeltaPro system using the time-correlated single-photon counting method (TCSPC) (Horiba Scientific, Glasgow, UK). This method measures fluorescence lifetimes from picoseconds to microseconds. The samples were excited with a violet (404 nm) pulsed laser diode (Horiba Scientic, Glasgow, UK). On the detector side, a 550 nm long-pass (LP) filter was used with compounds **1_A_**–**8_A_** and a 715 nm LP filter (Thorlabs, Newton, NJ, USA) with compounds **1_B_**–**8_B_**.

#### 3.3.4. Singlet Oxygen Quantum Yields

Singlet oxygen sensitization was determined from the relative intensity of the ^1^O_2_ emission band, which is centered around 1276 nm, using [Ru(bpy)_3_]^2+^ as the standard (Φ_Δ_ = 0.56 in aerated MeCN) [49]. Quantum yields (Φ_Δ_) were calculated in accordance with the actinometric method described by Equation (4), where *I* denotes the integration of the emission band, *A* is the solution UV-Vis absorption at the excitation wavelength, and *n* is the solvent’s refractive index (*n*^2^/*n*_s_^2^ = 1 here, as MeCN was used in both). The [Ru(bpy)_3_]^2+^ standard is denoted by the subscript *S*. It is generally desirable to run such measurements in MeCN because this solvent does not quench the ^1^O_2_ state (unlike water), and its common use in the literature facilitates comparisons.

(4) $$\begin{array}{c} \Phi_{\Delta }=\Phi_{\Delta ,S}\left(\frac{I}{I_{S}}\right)\left(\frac{A_{S}}{A}\right)\left(\frac{n^{2}}{n_{S}^{2}}\right) \end{array}$$

Emission spectra were measured on a PTI Quantamaster emission spectrometer equipped with a Hamamatsu R550942 near-infrared photomultiplier tube behind a 1000 nm long pass filter. Emission and excitation spectra were corrected for nonlinearities in lamp output and detector response. The longest wavelength in the excitation spectrum that maximized emission at 1276 nm was selected for the excitation wavelength. The emission spectra were collected over 1200–1350 nm and integrated with baseline correction. The values were generally reproducible within ±5%.

### 3.4. Log(D_o/w_) Measurements

Complexes **5**, **6**, and **7** were measured in the current study, whereas other compounds were measured in past work. Solutions of 1-octanol and buffer at pH 7.4 were mixed in a 1:1 ratio and stirred for 24 h before use to ensure the solutions were saturated with the corresponding solution. The procedure used to measure Log(D*_o/w_*) as a function of pH was a modified “shake flask” method that was deemed acceptable for use by measuring the Log(D*_o/w_*) at pH 7.4 of 5-fluorouracil and comparing those results with reported literature values [50]. As a general procedure, the ruthenium compound of interest (200 μM) was first dissolved in *n*-octanol saturated with buffer. A portion of this solution (5 mL) was then mixed with an equal volume of buffer saturated with *n*-octanol and gently stirred for 24 h at ambient temperature. Afterwards, an aliquot was removed from the aqueous phase, filtered, and the absorbance was measured via UV-Vis spectroscopy to determine the concentration in the aqueous phase. Similarly, an aliquot was removed from the organic phase, filtered, and the absorbance was measured via UV-Vis spectroscopy to determine the concentration in the organic phase. This allowed the calculation of a Log(D*_o/w_*) value (Equation (5)). All measurements were performed in at least quadruplicate with the average Log(D*_o/w_*) reported.

Log(D*_o/w_*) = Log ([Ru]_Org_/[Ru]_Aq_) (5)

### 3.5. Cellular Viability Assays

The current work used MCF7 with complex **7_A_**, and all other cell studies in Table 1 are previously published. Breast epithelial adenocarcinoma MCF7 cells (purchased from ATCC) were seeded at a density of 20,000 cells per well in 100 µL of Dulbecco’s modified Eagle medium supplemented with 10% fetal bovine serum in 96-well plates and incubated for 48 h to allow the cells to adhere to the plate. Complex **7_A_** was dissolved in DMSO and diluted in media to avoid a cytotoxic effect from DMSO on the cells. The final concentration of DMSO was set to less than 1% (*v/v*). The cells were treated with 100 µL of serially diluted compounds and incubated for 48 h in the dark. The cells were then washed with phosphate-buffered saline (200 µL × 3) and irradiated for two hours with white light (STASUN 200W LED Flood Light, 100–256 V, 20,000 lm, 40,000 lux, irradiance: 40 mW cm^−2^, total fluence: 288 J cm^−2^). The cells were then provided with 100 µL of fresh media per well and incubated in the dark for 24 h. The cytotoxic effects of the compounds were measured using a Cell Counting Kit-8 according to the manufacturer’s protocol (Enzo Life Sciences). The EC_50_ of each cell line was determined using a nonlinear regression fit of the dose-response curve (using GraphPad (GraphPad Software, Boston, MA, USA) and Minitab 19 (Minitab, LLC, Chicago IL, USA) using Equation (6):

(6) $$A_{obs}=A_{min}+\frac{A_{max}-A_{min}}{1+10^{\left(logEC50-\log\left[Ru\right]\right)}}\;$$

In the above formula, “*A_min_*” and “*A_max_*” are the minimum and maximum absorbances where the curve reaches a plateau and [Ru] is the concentration of **7_A_**.

## 4. Conclusions

Eight protic ruthenium complexes have been studied as potential light activated anticancer agents, and three complexes (**3**, **4**, and **8**) exhibited significant phototoxicity indices (Table 1). Past work has suggested that singlet oxygen formation rather than photodissociation correlates with the observed photocytotoxicity [11,26]. The singlet oxygen quantum yields for these complexes have also been studied and have revealed that several complexes are competent at singlet oxygen formation (**1**, **2**, **5**, **6**, and **7**) yet are not photocytotoxic due to poor cellular uptake. The newly synthesized and crystallized complex **7_A_** follows the trend exhibited by **5_A_** and **6_A_**, namely that all of these complexes produce singlet oxygen efficiently but poor uptake is predicted by log(D_o/w_) values of 0.8 and lower. Only with the lipophilic BPhen ligand (log(D_o/w_) > 3) does the 4,4′-dhbp ligand lead to significant photocytotoxicity in [(BPhen)_2_Ru(4,4′-dhbp)]Cl_2_, **8_A_**. Photoluminescence studies including photoluminescence quantum yields and lifetimes were measured for all sixteen complexes (**1_A_**–**8_A_**, **1_B_**–**8_B_**) in deaerated acetonitrile. This reveals several trends that correlate with the singlet oxygen quantum yields. In complexes **1**–**4**, both the OH bearing (**A** forms) and O^−^ bearing (**B** forms) variants undergo multiexponential decay processes with τ values less than 1 μs (Figures S14–S21). The thermally accessible ^3^MC state may provide a pathway for decay which can lead to photodissociation, as shown in our past work (Figure 1a,b) [11,26]. Complexes **5_B_**–**8_B_** all exhibit biexponential decay with τ values less than 1 μs, but in this case, electron density on the O^−^ groups is transferred to a spectator ligand via the ^3^LLCT state which serves to quench luminescence. Complexes **5_A_**–**8_A_** undergo monoexponential decay with exceptionally long lifetimes that increase with π expansion (as high as 3.45 μs with an 18.7% PLQY for **8_A_**). This work has correlated the high singlet oxygen quantum yields for **5_A_**–**8_A_** with a long lifetime for the excited state. Together, this explains the unique photocytotoxicity for **8**, which likely involves **8** (in multiple protonation states) entering the cells, and while both **8_A_** and **8_B_** can generate singlet oxygen, our work suggests that **8_A_** is much more efficient at this process due to a long-lived excited state.

## Figures and Tables

**Table 1:** A comparison of the cellular studies performed for light activated toxicity as correlated with photophysical properties (quantum yields). Here we include a select number of cellular phototoxicity studies. Log(D*_o/w_*) values are provided as a measure of relative hydrophilicity vs. lipophilicity of the compounds. N.d. = not determined.

Compound	Cell Line ^a^	EC_50_light_ (μM)	EC_50_dark_ (μM)	PI	p*K*_a_ avg	log(D*_o/w_*) at pH 7.4	Φ_PS_ (%) ^d^	Φ_Δ_ (%)	Ref.
**1_A_**	MCF7	>500 ^b^	>500	~1	6.3	1.4(1)	0.58	4.1 ^g^	[11,25], [28,29]
**1_B_**							0.12	18 ^g^	
**2_A_**	MCF7	180 ^b^	490	2.8	6.0(1)	1.6(1)	0.20	4.8 ^g^	[11,28,29]
**2_B_**							3.6 × 10^−3^	87 ^g^	
**3_A_**	MCF7	4.1 ^b^	490	120	5.9(1)	1.8(1)	0.1	4.8 ^g^	[11,28,29]
**3_B_**							2.2 × 10^−2^	48 ^g^	
**4_A_**	MCF7	2.0 ^c^	18	8.9	5(1)	>3	^e^	5 ^h^	[26]
**4_B_**								<1 ^h^	
**5_A_**	HeLa	>100 ^b^	>100	~1	n.d.	−1.2(2)	^f^	57 ^h^	This work, [25]
**5_B_**								4 ^h^	
**6_A_**	MCF7	194 ^b^	>300	1.5	6.01(8)	−0.1(2)	^f^	62 ^h^	This work, [28]
**6_B_**								16, 19 ^h,i^	
**7_A_**	MCF7	>100 ^b^	>100	~1	n.d.	0.8(2)	^f^	76 ^h^	This work
**7_B_**								15 ^h,j^	
**8_A_**	MCF7	0.5 ^c^	>100	>200	5.9(3)	>3	^f^	68 ^h^	[26]
**8_B_**								19 ^h^	

^a^ The cell studies for these compounds are performed in media at pH 7.4 to 7.5. Thus, based upon published p*K*_a_ values, mostly the B form is present in the cell studies, although the compound is initially given to cells in the A form. The form listed here (A or B) corresponds to which form was used to measure singlet oxygen formation or photosubstitution in columns 8 and 9. ^b^ Light fluence and illumination area not reported. Irradiated for 60 min with blue light at λ = 450 nm. ^c^ Irradiated with white LED light with significant amounts of blue frequencies (λ = 450 nm). Dosage of light = 40 mWcm^−2^ for 120 min, 288 Jcm^−2^. ^d^ For **1_A_**, **2_A_**, and **3_A_**, the Φ_PS_ was measured in aqueous buffer at pH 5.0 because this gives mostly the OH bearing A form based on the p*K*_a_ values. For **1_B_**, **2_B_**, and **3_B_**, the Φ_PS_ was measured in aqueous buffer at pH 7.5 because this gives mostly the O^−^ bearing B form based on the p*K*_a_ values. ^e^ Not reported but photosubstitution is observed. ^f^ Not reported and photosubstitution has not been observed for this system. ^g^ Measured in CD_3_OD. ^h^ Measured in CH_3_CN. ^i^ Values of Φ_Δ_ = 16% and 19% were measured with excitation at 510 nm and 471 nm, respectively. ^j^ Values of Φ_Δ_ = 15% and 14% were measured with excitation at 465 nm and 517 nm, respectively.

**Table 2:** Selected bond lengths and angles for **7_A_** from single crystal X-ray diffraction.

Bond Lengths (Å)		Bond Angles (°)	
Ru1-N1	2.053(2)	N1-Ru1-N2	78.38(8)
Ru1-N2	2.060(2)	N1-Ru1-N3	96.24(8)
Ru1-N3	2.060(2)	N1-Ru1-N4	89.49(9)
Ru1-N4	2.061(2)	N1-Ru1-N5	172.40(9)
Ru1-N5	2.071(2)	N2-Ru1-N4	97.68(9)
Ru1-N6	2.051(2)	N2-Ru1-N5	95.68(9)
C3-O1	1.336(3)	N3-Ru1-N2	174.24(9)
C8-O2	1.341(3)	N3-Ru1-N4	80.09(9)
		N3-Ru1-N5	89.86(9)
		N4-Ru1-N5	96.06(9)
		N6-Ru1-N1	95.05(9)
		N6-Ru1-N2	89.96(9)
		N6-Ru1-N3	92.58(9)
		N6-Ru1-N4	171.78(9)
		N6-Ru1-N5	80.09(9)

**Table 3:** Photoluminescence quantum yields, measured monoexponential lifetimes and calculated radiative lifetimes of the acidic forms of [(bpy)_3_Ru]Cl_2_ and **5_A_**–**8_A_**. These were measured in dry acetonitrile from a solvent purification system and prepared under nitrogen.

Compound	Φ_PL_	Measured Monoexponential Lifetime, τ_meas_ (μs)	Calculated Radiative Lifetime, τ_rad_ (µs)
**[(bpy)_3_Ru]Cl_2_**	9.4%	0.882	9.4
**5_A_**	5.3%	0.538	10.2
**6_A_**	12.8%	1.81	14.1
**7_A_**	15.3%	1.682	11.0
**8_A_**	18.7%	3.45	18.5

## Data Availability

Data is available upon request.

## Supplementary Materials

The following supporting information can be downloaded at: https://www.mdpi.com/article/10.3390/ijms24065980/s1

## References

[1] Fiedler E.C., Hemann M.T. (2019). Aiding and Abetting: How the Tumor Microenvironment Protects Cancer from Chemotherapy. Annu. Rev. Cancer Biol..

[2] Monro S., Colón K.L., Yin H., Roque J., Konda P., Gujar S., Thummel R.P., Lilge L., Cameron C.G., McFarland S.A. (2019). Transition Metal Complexes and Photodynamic Therapy from a Tumor-Centered Approach: Challenges, Opportunities, and Highlights from the Development of TLD1433. Chem. Rev..

[3] McFarland S.A., Mandel A., Dumoulin-White R., Gasser G. (2020). Metal-based photosensitizers for photodynamic therapy: The future of multimodal oncology?. Curr. Opin. Chem. Biol..

[4] Fong J., Kasimova K., Arenas Y., Kaspler P., Lazic S., Mandel A., Lilge L. (2015). A novel class of ruthenium-based photosensitizers effectively kills in vitro cancer cells and in vivo tumors. Photochem. Photobiol. Sci..

[5] Papish E.T., Oladipupo O. (2022). Factors that Influence Singlet Oxygen Formation vs. Ligand Substitution for Light Activated Ruthenium Anticancer Compounds. Curr. Opin. Chem. Biol..

[6] Hachey A.C., Havrylyuk D., Glazer E.C. (2021). Biological activities of polypyridyl-type ligands: Implications for bioinorganic chemistry and light-activated metal complexes. Curr. Opin. Chem. Biol..

[7] Roque J., Havrylyuk D., Barrett P.C., Sainuddin T., McCain J., Colón K., Sparks W.T., Bradner E., Monro S., Heidary D. (2020). Strained, Photoejecting Ru(II) Complexes that are Cytotoxic Under Hypoxic Conditions. Photochem. Photobiol..

[8] Chen Y., Bai L., Zhang P., Zhao H., Zhou Q. (2021). The Development of Ru(II)-Based Photoactivated Chemotherapy Agents. Molecules.

[9] Lanquist A.P., Gupta S., Al-Afyouni K.F., Al-Afyouni M., Kodanko J.J., Turro C. (2021). Trifluoromethyl substitution enhances photoinduced activity against breast cancer cells but reduces ligand exchange in Ru(II) complex. Chem. Sci..

[10] White J.K., Schmehl R.H., Turro C. (2017). An overview of photosubstitution reactions of Ru(II) imine complexes and their application in photobiology and photodynamic therapy. Inorg. Chim. Acta.

[11] Qu F., Lamb R.W., Cameron C.G., Park S., Oladipupo O., Gray J.L., Xu Y., Cole H.D., Bonizzoni M., Kim Y. (2021). Singlet Oxygen Formation vs Photodissociation for Light-Responsive Protic Ruthenium Anticancer Compounds: The Oxygenated Substituent Determines Which Pathway Dominates. Inorg. Chem..

[12] Verma S., Kar P., Das A., Ghosh H.N. (2011). Photophysical properties of ligand localized excited state in ruthenium(ii) polypyridyl complexes: A combined effect of electron donor–acceptor ligand. Dalton Trans..

[13] Toupin N.P., Nadella S., Steinke S.J., Turro C., Kodanko J.J. (2020). Dual-Action Ru(II) Complexes with Bulky π-Expansive Ligands: Phototoxicity without DNA Intercalation. Inorg. Chem..

[14] Reichardt C., Monro S., Sobotta F.H., Colón K.L., Sainuddin T., Stephenson M., Sampson E., Roque J., Yin H., Brendel J.C. (2019). Predictive Strength of Photophysical Measurements for in Vitro Photobiological Activity in a Series of Ru(II) Polypyridyl Complexes Derived from π-Extended Ligands. Inorg. Chem..

[15] Plaetzer K., Krammer B., Berlanda J., Berr F., Kiesslich T. (2009). Photophysics and photochemistry of photodynamic therapy: Fundamental aspects. Lasers Med. Sci..

[16] Van Straten D., Mashayekhi V., De Bruijn H.S., Oliveira S., Robinson D.J. (2017). Oncologic Photodynamic Therapy: Basic Principles, Current Clinical Status and Future Directions. Cancers.

[17] Soupart A., Alary F., Heully J.-L., Elliott P.I.P., Dixon I.M. (2020). Recent progress in ligand photorelease reaction mechanisms: Theoretical insights focusing on Ru(II) 3MC states. Coord. Chem. Rev..

[18] Loftus L.M., Rack J.J., Turro C. (2020). Photoinduced ligand dissociation follows reverse energy gap law: Nitrile photodissociation from low energy ^3^MLCT excited states. Chem. Commun..

[19] Mukuta T., Tanaka S.I., Inagaki A., Koshihara S.-Y., Onda  K. (2016). Direct Observation of the Triplet Metal-Centered State in [Ru(bpy)3]2+ Using Time-Resolved Infrared Spectroscopy. ChemistrySelect.

[20] Havrylyuk D., Stevens K., Parkin S., Glazer E.C. (2020). Toward Optimal Ru(II) Photocages: Balancing Photochemistry, Stability, and Biocompatibility Through Fine Tuning of Steric, Electronic, and Physiochemical Features. Inorg. Chem..

[21] Loftus L.M., Al-Afyouni K.F., Turro C. (2018). New RuII Scaffold for Photoinduced Ligand Release with Red Light in the Photodynamic Therapy (PDT) Window. Chem.—A Eur. J..

[22] Lameijer L.N., Ernst D., Hopkins S.L., Meijer M.S., Askes S.H.C., Le Dévédec S.E., Bonnet S. (2017). A Red-Light-Activated Ruthenium-Caged NAMPT Inhibitor Remains Phototoxic in Hypoxic Cancer Cells. Angew. Chem. Int. Ed..

[23] Li A., White J.K., Arora K., Herroon M.K., Martin P.D., Schlegel H.B., Podgorski I., Turro C., Kodanko J.J. (2016). Selective Release of Aromatic Heterocycles from Ruthenium Tris(2-pyridylmethyl)amine with Visible Light. Inorg. Chem..

[24] Cuello-Garibo J.-A., Meijer M.S., Bonnet S. (2017). To cage or to be caged? The cytotoxic species in ruthenium-based photoactivated chemotherapy is not always the metal. Chem. Commun..

[25] Hufziger K.T., Thowfeik F.S., Charboneau D.J., Nieto I., Dougherty W.G., Kassel W.S., Dudley T.J., Merino E.J., Papish E.T., Paul J.J. (2014). Ruthenium Dihydroxybipyridine Complexes are Tumor Activated Prodrugs due to Low pH and Blue Light Induced Ligand Release. J. Inorg. Biochem..

[26] Oladipupo O., Brown S.R., Lamb R.W., Gray J.L., Cameron C.G., DeRegnaucourt A.R., Ward N.A., Hall J.F., Xu Y., Petersen C.M. (2022). Light-responsive and Protic Ruthenium Compounds Bearing Bathophenanthroline and Dihydroxybipyridine Ligands Achieve Nanomolar Toxicity towards Breast Cancer Cells. Photochem. Photobiol..

[27] Qu F., Martinez K., Arcidiacono A.M., Park S., Zeller M., Schmehl R.H., Paul J.J., Kim Y., Papish E.T. (2018). Sterically demanding methoxy and methyl groups in ruthenium complexes lead to enhanced quantum yields for blue light triggered photodissociation. Dalton Trans..

[28] Qu F., Park S., Martinez K., Gray J.L., Thowfeik F.S., Lundeen J.A., Kuhn A.E., Charboneau D.J., Gerlach D.L., Lockart M.M. (2017). Ruthenium Complexes are pH-Activated Metallo Prodrugs (pHAMPs) with Light-Triggered Selective Toxicity Toward Cancer Cells. Inorg. Chem..

[29] Park S., Gray J.L., Altman S.D., Hairston A.R., Beswick B.T., Kim Y., Papish E.T. (2020). Cellular uptake of protic ruthenium complexes is influenced by pH dependent passive diffusion and energy dependent efflux. J. Inorg. Biochem..

[30] Reichardt C., Sainuddin T., Wächtler M., Monro S., Kupfer S., Guthmuller J., Gräfe S., McFarland S., Dietzek B. (2016). Influence of Protonation State on the Excited State Dynamics of a Photobiologically Active Ru(II) Dyad. J. Phys. Chem. A..

[31] Tardito S., Bassanetti I., Bignardi C., Elviri L., Tegoni M., Mucchino C., Bussolati O., Franchi-Gazzola R., Marchiò L. (2011). Copper Binding Agents Acting as Copper Ionophores Lead to Caspase Inhibition and Paraptotic Cell Death in Human Cancer Cells. J. Am. Chem. Soc..

[32] Zeng L., Chen Y., Huang H., Wang J., Zhao D., Ji L., Chao H. (2015). Cyclometalated Ruthenium(II) Anthraquinone Complexes Exhibit Strong Anticancer Activity in Hypoxic Tumor Cells. Chem. —A Eur. J..

[33] Tabrizi L., Chiniforoshan H. (2016). New Ru IIpincer complexes: Synthesis, characterization and biological evaluation for photodynamic therapy. Dalton Trans..

[34] Mehanna S., Mansour N., Audi H., Bodman-Smith K., Mroueh M.A., Taleb R.I., Daher C.F., Khnayzer R.S. (2019). Enhanced cellular uptake and photochemotherapeutic potential of a lipophilic strained Ru( ii) polypyridyl complex. RSC Adv..

[35] Gray J.L. (2020). Light-Activated Protic Ruthenium Anticancer Compounds: Structure Function Relationships and Determining Which Factors Influence Toxicity. Ph.D. Thesis.

[36] DePasquale J., Nieto I., Reuther L.E., Herbst-Gervasoni C.J., Paul J.J., Mochalin V., Zeller M., Thomas C.M., Addison A.W., Papish E.T. (2013). Iridium Dihydroxybipyridine Complexes Show That Ligand Deprotonation Dramatically Speeds Rates of Catalytic Water Oxidation. Inorg. Chem..

[37] Jeffrey G.A. (2003). Hydrogen-Bonding: An update. Crystallogr. Rev..

[38] Yao W., Das S., DeLucia N.A., Qu F., Boudreaux C.M., Vannucci A.K., Papish E.T. (2020). Determining the Catalyst Properties That Lead to High Activity and Selectivity for Catalytic Hydrodeoxygenation with Ruthenium Pincer Complexes. Organometallics.

[39] Yao W., DeRegnaucourt A.R., Shrewsbury E.D., Loadholt K.H., Silprakob W., Brewster T.P., Papish E.T. (2020). Reinvestigating Catalytic Alcohol Dehydrogenation with an Iridium Dihydroxybipyridine Catalyst. Organometallics.

[40] Brown R.T., Fletcher N.C., Danos L., Halcovitch N.R. (2019). A Tripodal Ruthenium(II) Polypyridyl Complex with pH Controlled Emissive Quenching. Eur. J. Inorg. Chem..

[41] Abrahamsson M., Jäger M., Kumar R.J., Österman T., Persson P., Becker H.-C., Johansson O., Hammarström L. (2008). Bistridentate Ruthenium(II)polypyridyl-Type Complexes with Microsecond 3MLCT State Lifetimes: Sensitizers for Rod-Like Molecular Arrays. J. Am. Chem. Soc..

[42] Hidayatullah A.N., Wachter E., Heidary D.K., Parkin S., Glazer E.C. (2014). Photoactive Ru(II) Complexes With Dioxinophenanthroline Ligands Are Potent Cytotoxic Agents. Inorg. Chem..

[43] Norris M.R., Concepcion J.J., Glasson C.R.K., Fang Z., Lapides A.M., Ashford D.L., Templeton J.L., Meyer T.J. (2013). Synthesis of Phosphonic Acid Derivatized Bipyridine Ligands and Their Ruthenium Complexes. Inorg. Chem..

[44] Sheldrick G.M. (2015). Crystal structure refinement with SHELXL. Acta Cryst..

[45] Dolomanov O.V., Bourhis L.J., Gildea R.J., Howard J.A.K., Puschmann H. (2009). OLEX2: A complete structure solution, refinement and analysis program. J. Appl. Crystallogr..

[46] Sheldrick G.M. (2008). A short history of SHELX. Acta Cryst..

[47] Suzuki K., Kobayashi A., Kaneko S., Takehira K., Yoshihara T., Ishida H., Shiina Y., Oishi S., Tobita S. (2009). Reevaluation of absolute luminescence quantum yields of standard solutions using a spectrometer with an integrating sphere and a back-thinned CCD detector. Phys. Chem. Chem. Phys..

[48] Ishida H., Tobita S., Hasegawa Y., Katoh R., Nozaki K. (2010). Recent advances in instrumentation for absolute emission quantum yield measurements. Coord. Chem. Rev..

[49] DeRosa M.C., Crutchley R.J. (2002). Photosensitized singlet oxygen and its applications. Coord. Chem. Rev..

[50] El Maghraby G.M.M., Williams A.C., Barry B.W. (2005). Drug interaction and location in liposomes: Correlation with polar surface areas. Int. J. Pharm..

