# Supplementary material for: Ruthenium Complexes with Protic Ligands: Influence of the Position of OH Groups and π Expansion on Luminescence and Photocytotoxicity
Source: Int J Mol Sci. 2023 Mar 22;24(6):5980. doi: 10.3390/ijms24065980 (PMC10053956; doi:10.3390/ijms24065980)

checkCIF/PLATON report

Structure factors have been supplied for datablock(s) jlg11-qu

THIS REPORT IS FOR GUIDANCE ONLY. IF USED AS PART OF A REVIEW PROCEDURE FOR PUBLICATION, IT SHOULD NOT REPLACE THE EXPERTISE OF AN EXPERIENCED CRYSTALLOGRAPHIC REFEREE.

No syntax errors found. CIF dictionary Interpreting this report

Datablock: jlg11-qu

Bond precision:	C-C = 0.0042 A		Wavelength=0.71073
Cell:	a=13.5194(2)	b=18.9737(2)	c=28.8521(4)
	alpha=90	beta=90	gamma=90
Temperature:	100 K		

	Calculated	Reported
Volume	7400.94(17)	7400.94(17)
Space group	P b c a	P b c a
Hall group	-P 2ac 2ab	-P 2ac 2ab
Moiety formula	C38 H28 N6 O6 Ru, 2(Cl), 3(H2 O)	C38 H28 N6 O6 Ru, 2(Cl), 3(H2 O)
Sum formula	C38 H34 Cl2 N6 O9 Ru	C38 H34 Cl2 N6 O9 Ru
Mr	890.68	890.68
Dx,g cm-3	1.599	1.599
Z	8	8
Mu (mm-1)	0.634	0.634
F000	3632.0	3632.0
F000’	3625.78	
h,k,lmax	19,27,41	19,27,41
Nref	11287	11227
Tmin,Tmax	0.926,0.955	0.936,1.000
Tmin’	0.912	

Correction method= # Reported T Limits: Tmin=0.936 Tmax=1.000
AbsCorr = GAUSSIAN

Data completeness= 0.995 Theta(max)= 30.508

R(reflections)= 0.0478(8573) wR2(reflections)= 0.1391(11227)

S = 1.032 Npar= 526

The following ALERTS were generated. Each ALERT has the format
test-name_ALERT_alert-type_alert-level.
Click on the hyperlinks for more details of the test.

Alert level C

```
PLAT220_ALERT_2_C Non-Solvent  Resd 1  C   Ueq(max)/Ueq(min) Range        3.1 Ratio
PLAT241_ALERT_2_C High   'MainMol' Ueq as Compared to Neighbors of         O6 Check
PLAT241_ALERT_2_C High   'MainMol' Ueq as Compared to Neighbors of        C31 Check
PLAT906_ALERT_3_C Large K Value in the Analysis of Variance ......      2.566 Check
PLAT911_ALERT_3_C Missing FCF Refl Between Thmin & STh/L=    0.600          3 Report
PLAT971_ALERT_2_C Check Calcd Resid. Dens.  3.09A   From Cl2             1.98 eA-3
PLAT975_ALERT_2_C Check Calcd Resid. Dens.  0.90A   From O9              0.43 eA-3
PLAT976_ALERT_2_C Check Calcd Resid. Dens.  0.62A   From O7             -0.53 eA-3
PLAT976_ALERT_2_C Check Calcd Resid. Dens.  0.48A   From O7             -0.53 eA-3
PLAT976_ALERT_2_C Check Calcd Resid. Dens.  0.78A   From O7             -0.49 eA-3
PLAT976_ALERT_2_C Check Calcd Resid. Dens.  0.44A   From O7             -0.49 eA-3
```

Alert level G

```
PLAT007_ALERT_5_G Number of Unrefined Donor-H Atoms ..............          8 Report
PLAT083_ALERT_2_G SHELXL Second Parameter in WGHT  Unusually Large      11.23 Why ?
PLAT301_ALERT_3_G Main Residue  Disorder ..............(Resd  1  )         2% Note
PLAT794_ALERT_5_G Tentative Bond Valency for Ru1       (III)     .       3.03 Info
PLAT910_ALERT_3_G Missing # of FCF Reflection(s) Below Theta(Min).          3 Note
PLAT912_ALERT_4_G Missing # of FCF Reflections Above STh/L=  0.600         54 Note
PLAT933_ALERT_2_G Number of OMIT Records in Embedded .res File ...          1 Note
PLAT978_ALERT_2_G Number C-C Bonds with Positive Residual Density.          6 Info
```

---

0 **ALERT level A** = Most likely a serious problem - resolve or explain
0 **ALERT level B** = A potentially serious problem, consider carefully
11 **ALERT level C** = Check. Ensure it is not caused by an omission or oversight
8 **ALERT level G** = General information/check it is not something unexpected

0 ALERT type 1 CIF construction/syntax error, inconsistent or missing data
12 ALERT type 2 Indicator that the structure model may be wrong or deficient
4 ALERT type 3 Indicator that the structure quality may be low
1 ALERT type 4 Improvement, methodology, query or suggestion
2 ALERT type 5 Informative message, check

It is advisable to attempt to resolve as many as possible of the alerts in all categories. Often the minor alerts point to easily fixed oversights, errors and omissions in your CIF or refinement strategy, so attention to these fine details can be worthwhile. In order to resolve some of the more serious problems it may be necessary to carry out additional measurements or structure refinements. However, the purpose of your study may justify the reported deviations and the more serious of these should normally be commented upon in the discussion or experimental section of a paper or in the "special_details" fields of the CIF. checkCIF was carefully designed to identify outliers and unusual parameters, but every test has its limitations and alerts that are not important in a particular case may appear. Conversely, the absence of alerts does not guarantee there are no aspects of the results needing attention. It is up to the individual to critically assess their own results and, if necessary, seek expert advice.

Publication of your CIF in IUCr journals

A basic structural check has been run on your CIF. These basic checks will be run on all CIFs submitted for publication in IUCr journals (*Acta Crystallographica*, *Journal of Applied Crystallography*, *Journal of Synchrotron Radiation*); however, if you intend to submit to *Acta Crystallographica Section C* or *E* or *IUCrData*, you should make sure that full publication checks are run on the final version of your CIF prior to submission.

Publication of your CIF in other journals

Please refer to the *Notes for Authors* of the relevant journal for any special instructions relating to CIF submission.

PLATON version of 03/05/2019; check.def file version of 29/04/2019

Datablock jlg11-qu - ellipsoid plot

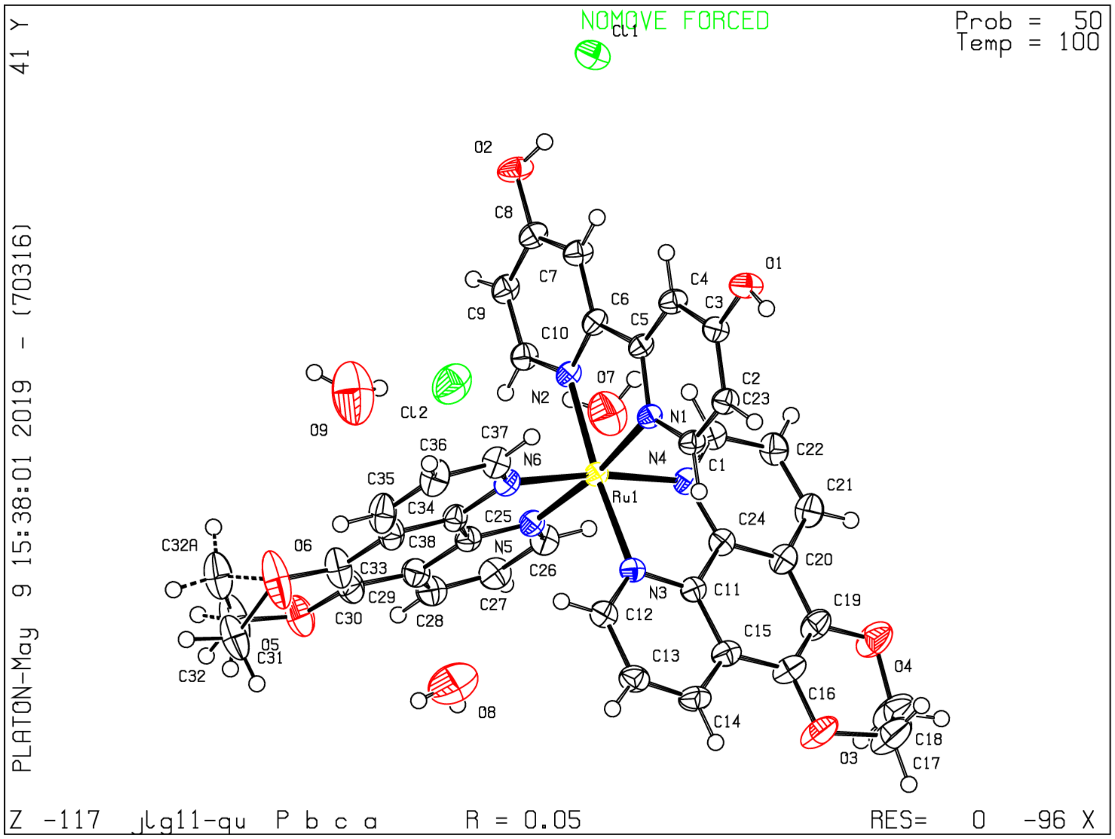

Supplement: Supplementary file 1 [file ijms-24-05980-s001.zip › JLG11-Qu_cifreport.pdf]
