# Supplementary material for: Ruthenium Complexes with Protic Ligands: Influence of the Position of OH Groups and π Expansion on Luminescence and Photocytotoxicity
Source: Int J Mol Sci. 2023 Mar 22;24(6):5980. doi: 10.3390/ijms24065980 (PMC10053956; doi:10.3390/ijms24065980)
Supplement: Supplementary file 1 [file ijms-24-05980-s001.zip › SI 2-21-23 acc all.pdf]

Supporting Information for

Ruthenium Complexes with Protic Ligands: Influence of the Position of OH Groups and Pi Expansion on Luminescence and Photocytotoxicity

Oladipupo, Olaitan E.;[I] Prescott, Meredith C.;[I] Blevins, Emily R.;[I] Gray, Jessica L.;[I] Cameron, Colin G.;[II] Qu, Fengrui;[I] Ward, Nicholas A.;[I] Pierce, Abigail L.;[I] Collinson, Elizabeth R.;[I] Hall, James Fletcher;[I] Park, Seungjo;[III, IV] Kim, Yonghyun;[III] McFarland, Sherri A.;[II] Fedin, Igor;[I,a] Papish, Elizabeth T.[I,b]

[I]The University of Alabama, Department of Chemistry and Biochemistry, Tuscaloosa, Alabama 35487, USA

[II]University of Texas Arlington, Department of Chemistry and Biochemistry, Arlington, Texas 76019, USA

[III]The University of Alabama, Department of Chemical and Biological Engineering, Tuscaloosa, Alabama 35487, USA

[IV]Present Address: Cytiva, 170 Locke Dr, Marlborough, MA 01752, USA.

[a]Corresponding author e-mail: ifedin@ua.edu, phone: 205-348-7020

[b]Corresponding author e-mail: etpapish@ua.edu, phone: 205-348-5822

Table of Contents

Characterization of $\mathbf{5_B}$, $\mathbf{6_B}$, $\mathbf{7_B}$, and $\mathbf{7_A}$……………..…………………………………….…3-7
UV-Vis Spectroscopy $\mathbf{5_A}$, $\mathbf{5_B}$, $\mathbf{6_A}$, $\mathbf{6_B}$, $\mathbf{7_A}$, and $\mathbf{7_B}$……………………………………………..8-10
Luminescence Studies $\mathbf{1_A}$, $\mathbf{1_B}$, $\mathbf{5_A}$, $\mathbf{5_B}$, $\mathbf{6_A}$, $\mathbf{6_B}$, $\mathbf{7_A}$, and $\mathbf{7_B}$ …………….................................11-14
Photoluminescence (PL) Dynamics of $\mathbf{1_A}$-$\mathbf{8_A}$, $\mathbf{1_B}$-$\mathbf{8_B}$..….…………………………………15-23
Summary of Photophysics for $\mathbf{1_A}$-$\mathbf{8_A}$, $\mathbf{1_B}$-$\mathbf{8_B}$ ……………………………………………….…….. 24
Single Crystal X-Ray Diffraction Data for $\mathbf{7_A}$ ………………..………..................................25

Characterization of the Complexes.

Complex 5_B

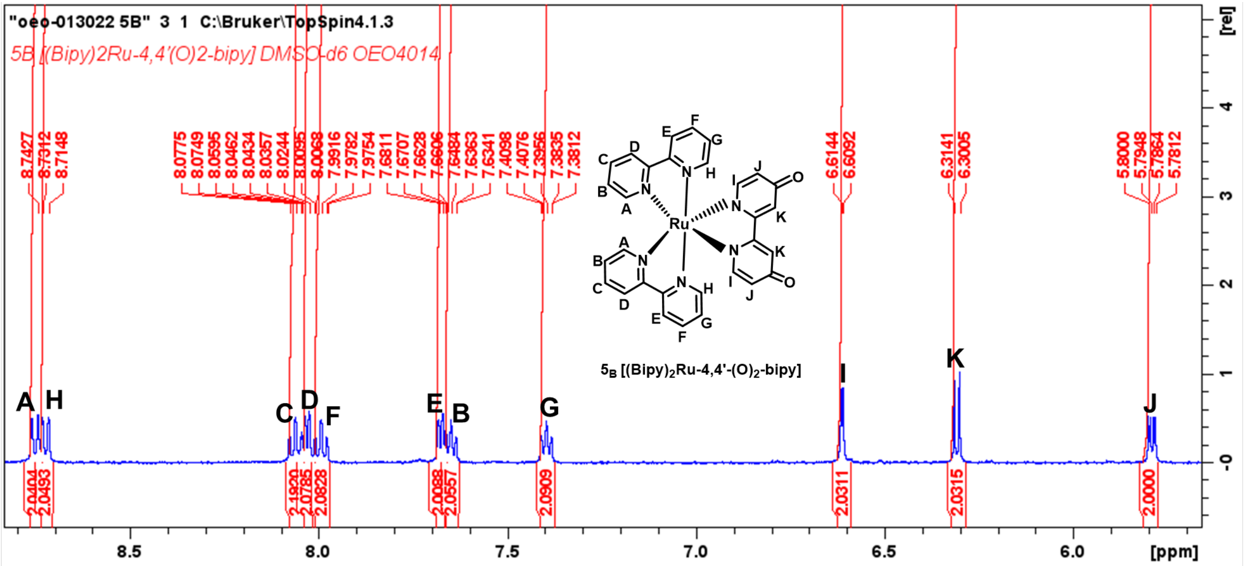

Figure S1. 500 MHz ^{1}HNMR (zoom of the aromatic region) spectrum of $\mathbf{5_B}$ in DMSO-d_6 at room temperature.

Complex 6_B

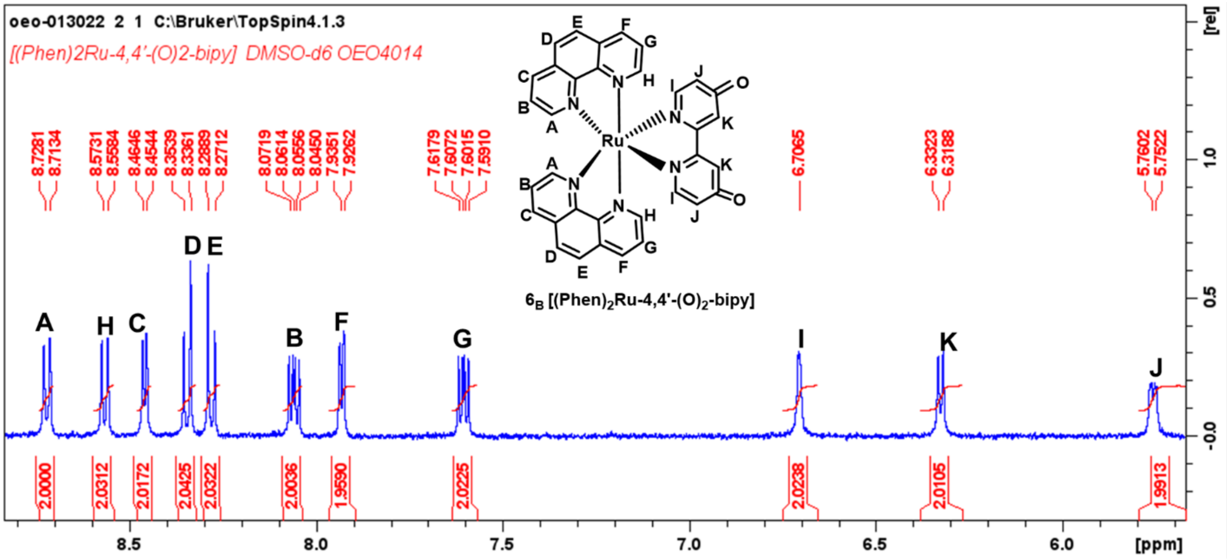

Figure S2. 500 MHz ^{1}HNMR (zoom of the aromatic region) spectrum of $\mathbf{6_B}$ in DMSO-d_6 at room temperature.

Complex 7_B

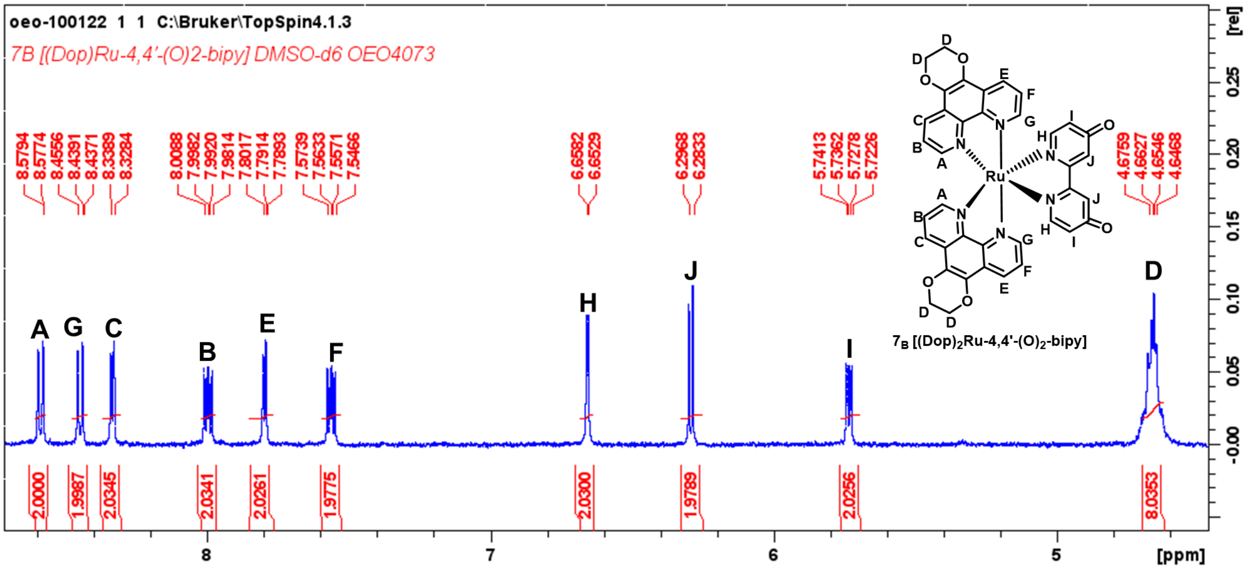

Figure S3. 500 MHz ^{1}HNMR spectrum of **7_B** in DMSO-d_6 at room temperature.

Complex 7_A

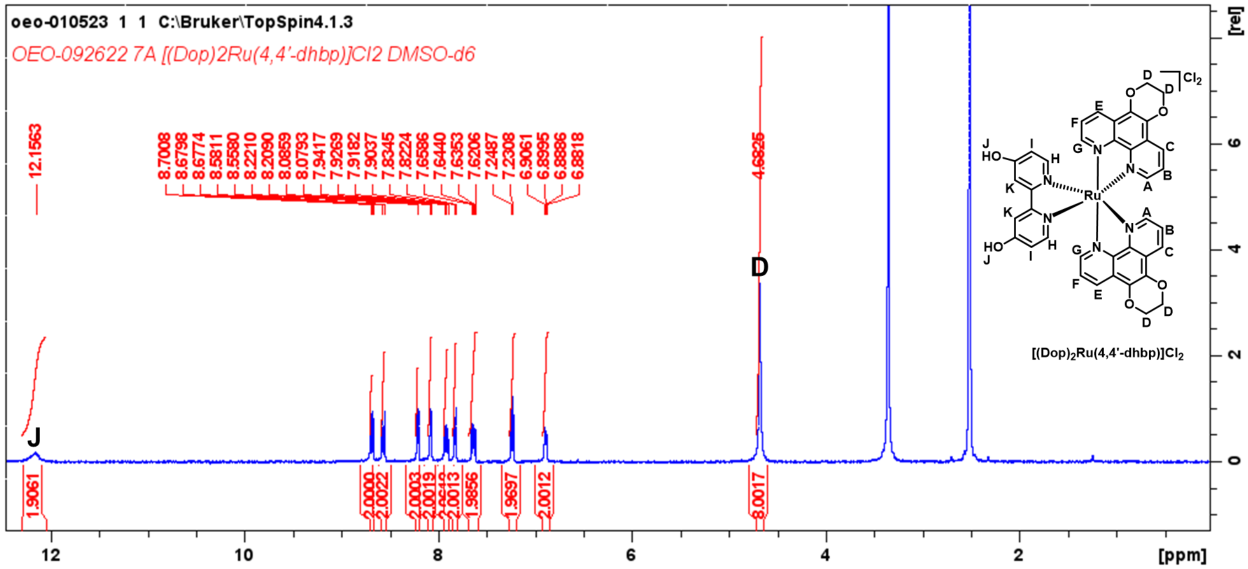

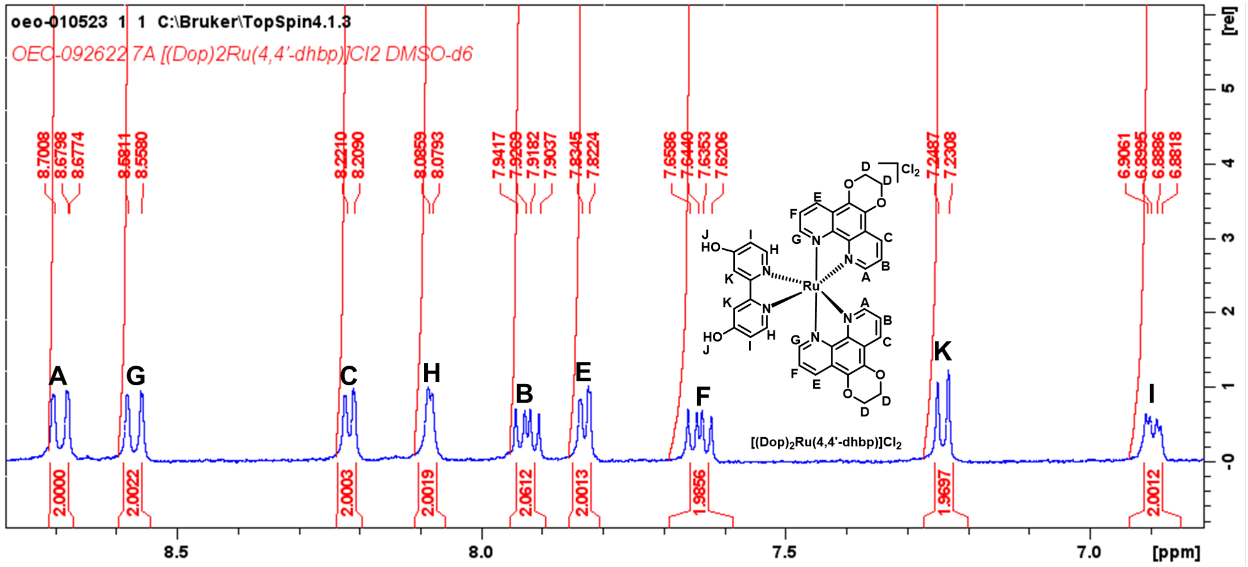

Figure S4. 500 MHz ^{1}HNMR spectrum of $\mathbf{7_A}$ in DMSO-d$_6$ at room temperature. Top: Full spectrum. Bottom: Zoom in of the aromatic region

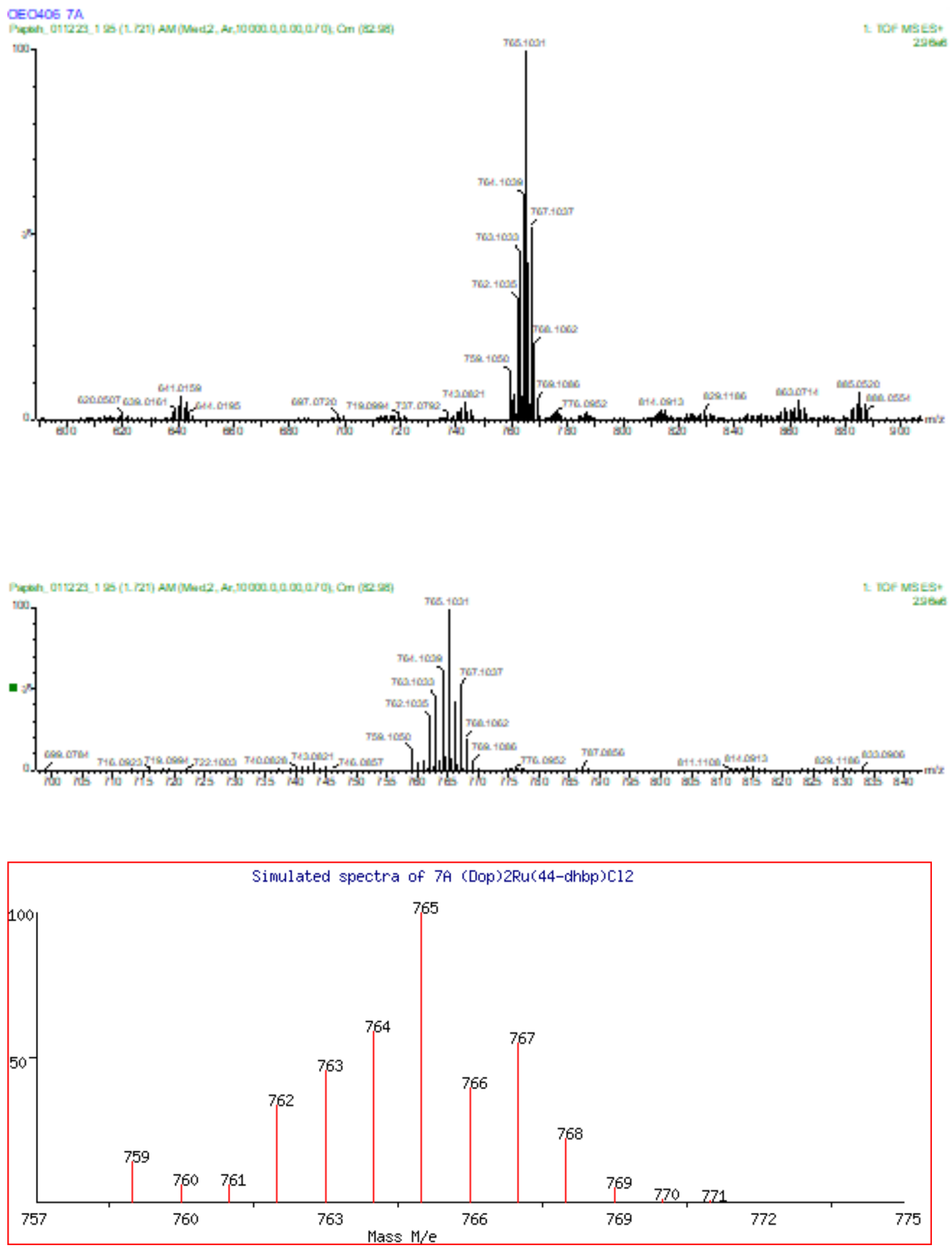

Figure S5. Top: High resolution ESI-MS of [(dop)$_2$Ru(4,4′-dhbp)]Cl$_2$ (**7$_A$**). The peak at m/z 765.1031 (calculated: 765.1036) corresponds to $C_{38}H_{27}N_6O_6Ru$ [M-H]$^+$. **Middle**: Zoomed view to show the isotopic distribution. **Bottom**: Simulated spectra of **7$_A$** [M-H]$^+$.

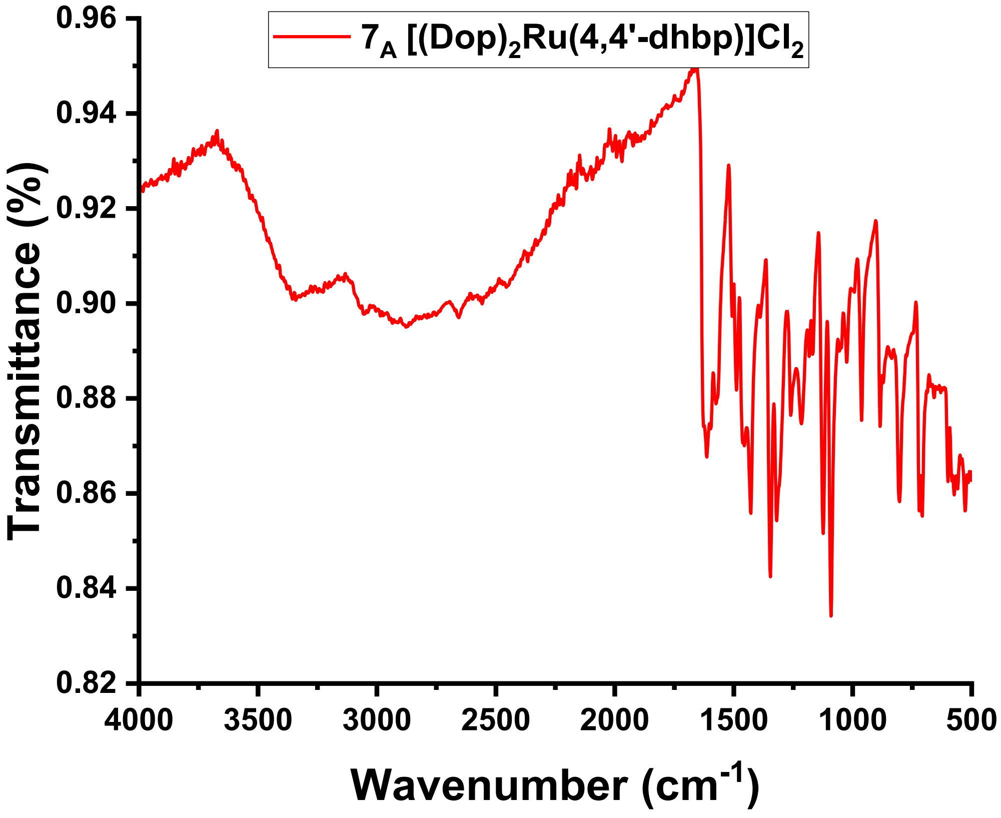

Figure S6. IR spectrum of $\mathbf{7_A}$. The observed vibrations include (in cm^{-1}): 3069, 2643, 1617, 1570, 1486, 1464, 1434.

UV-Vis Spectroscopy (Selected complexes are shown here, those not included were published previously)

Complexes 5_A and 5_B:

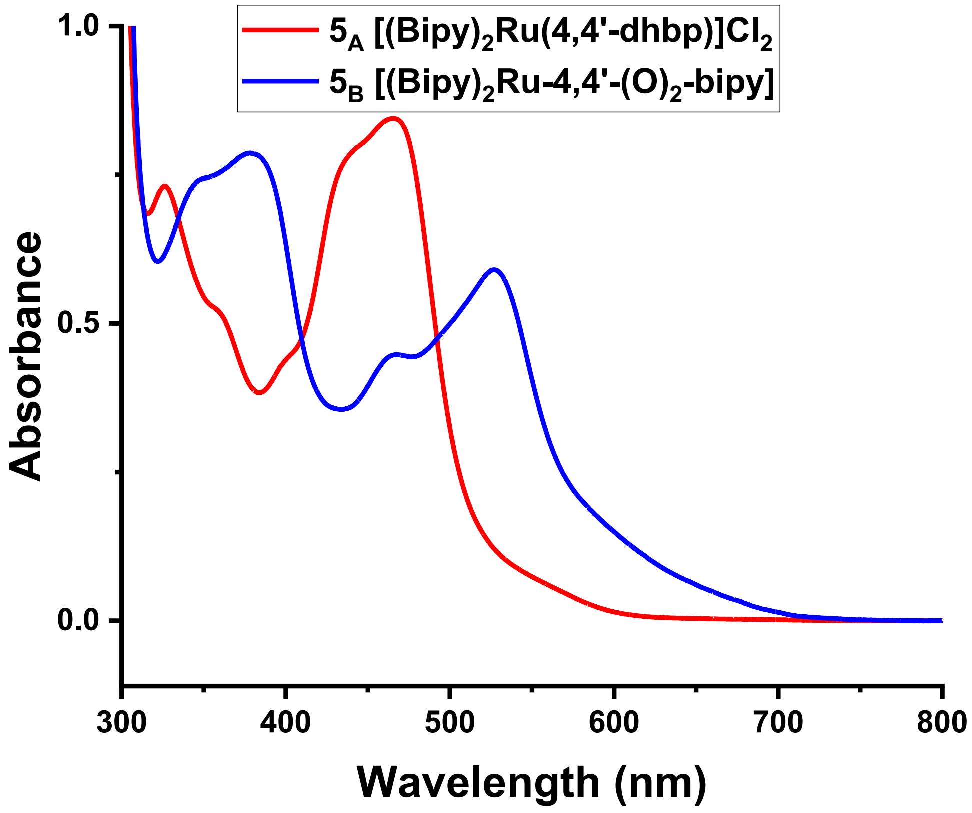

Figure S7. Overlaid UV-Vis spectra of **5_A** and **5_B** in acetonitrile at room temperature

Complexes 6_A and 6_B

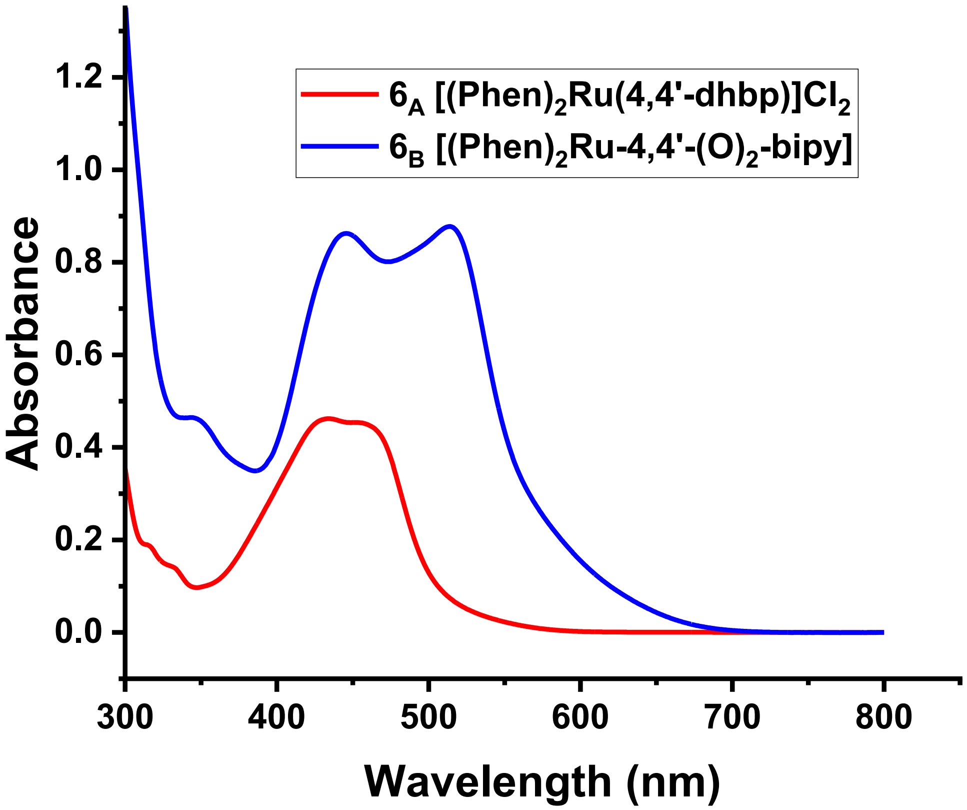

Figure S8. Overlaid UV-Vis spectra of $\mathbf{6_A}$ and $\mathbf{6_B}$ in acetonitrile at room temperature

Complexes 7_A and 7_B

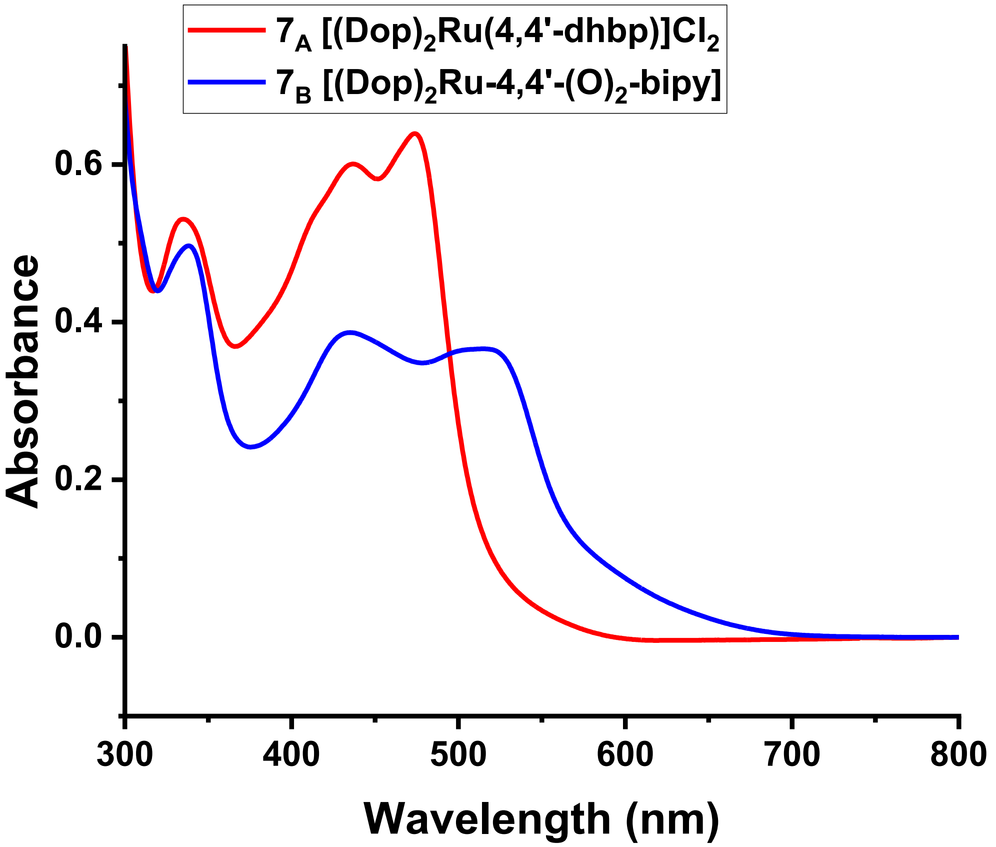

Figure S9. Overlaid UV-Vis spectra of $\mathbf{7_A}$ and $\mathbf{7_B}$ in acetonitrile at room temperature

.

Luminescence Studies (Selected complexes are shown here, those not included were published previously)

Complexes 1_A and 1_B

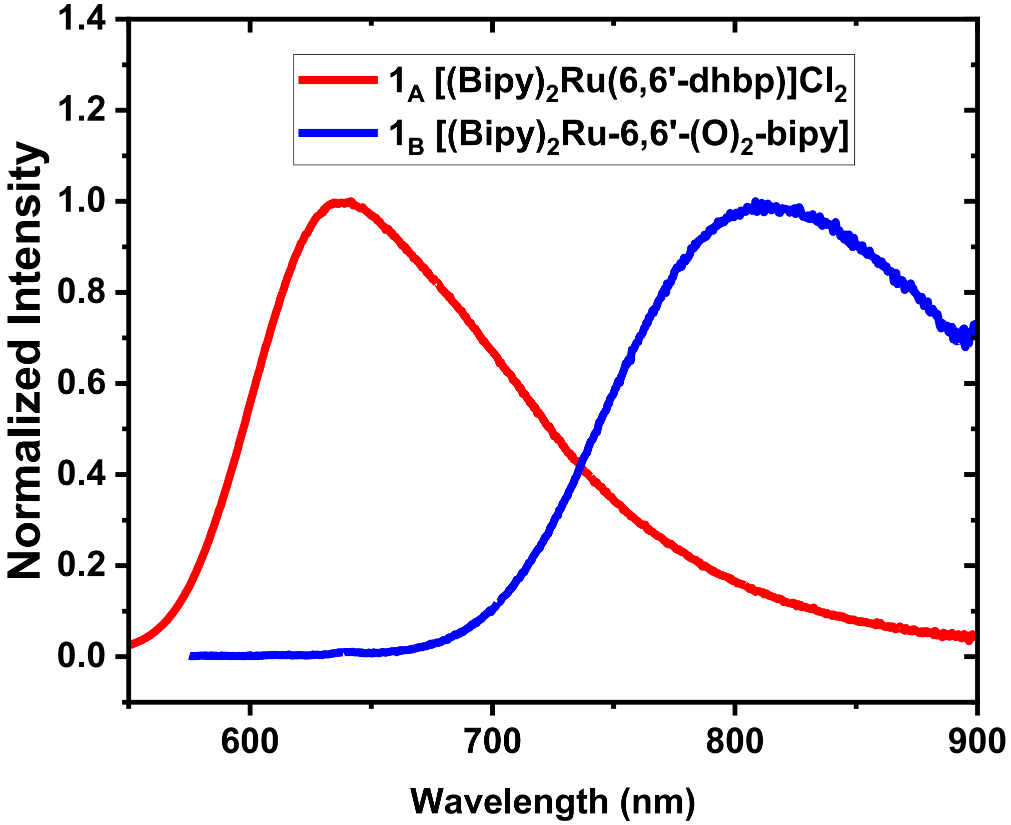

Figure S10. Room temperature overlaid photoluminescence spectra of $\mathbf{1_A}$ and $\mathbf{1_B}$ in acetonitrile

Complexes 5_A and 5_B

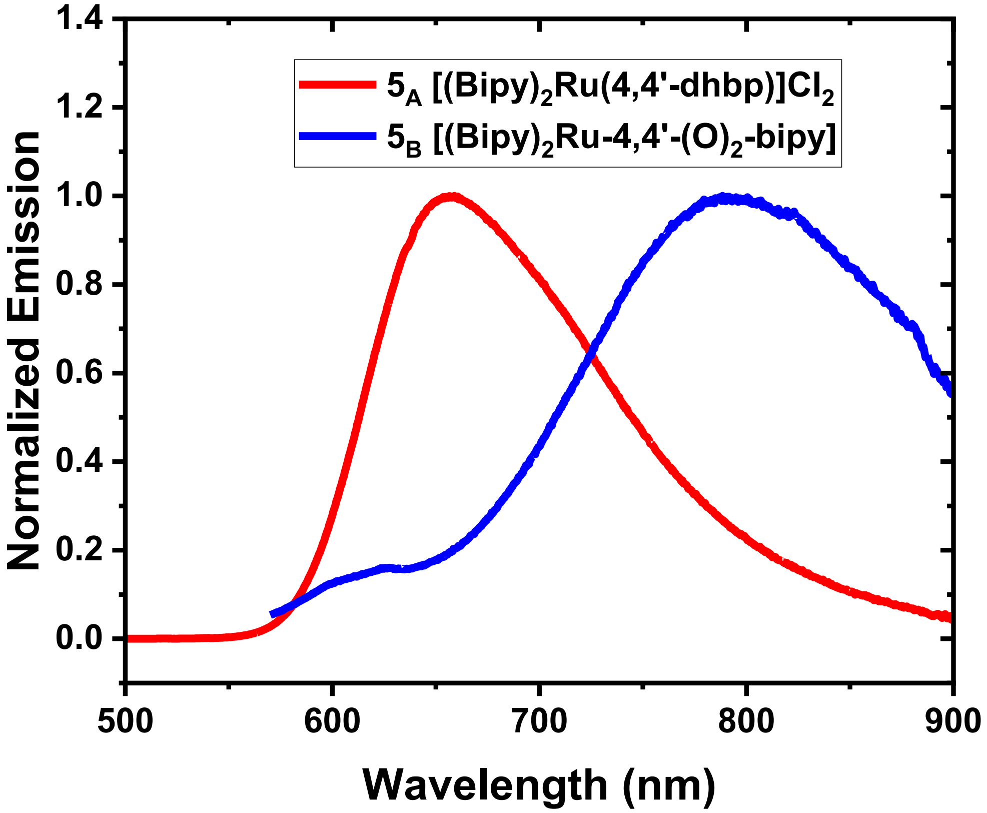

Figure S11: Room temperature overlaid photoluminescence spectra of $\mathbf{5_A}$ and $\mathbf{5_B}$ in acetonitrile

Complexes 6_A and 6_B

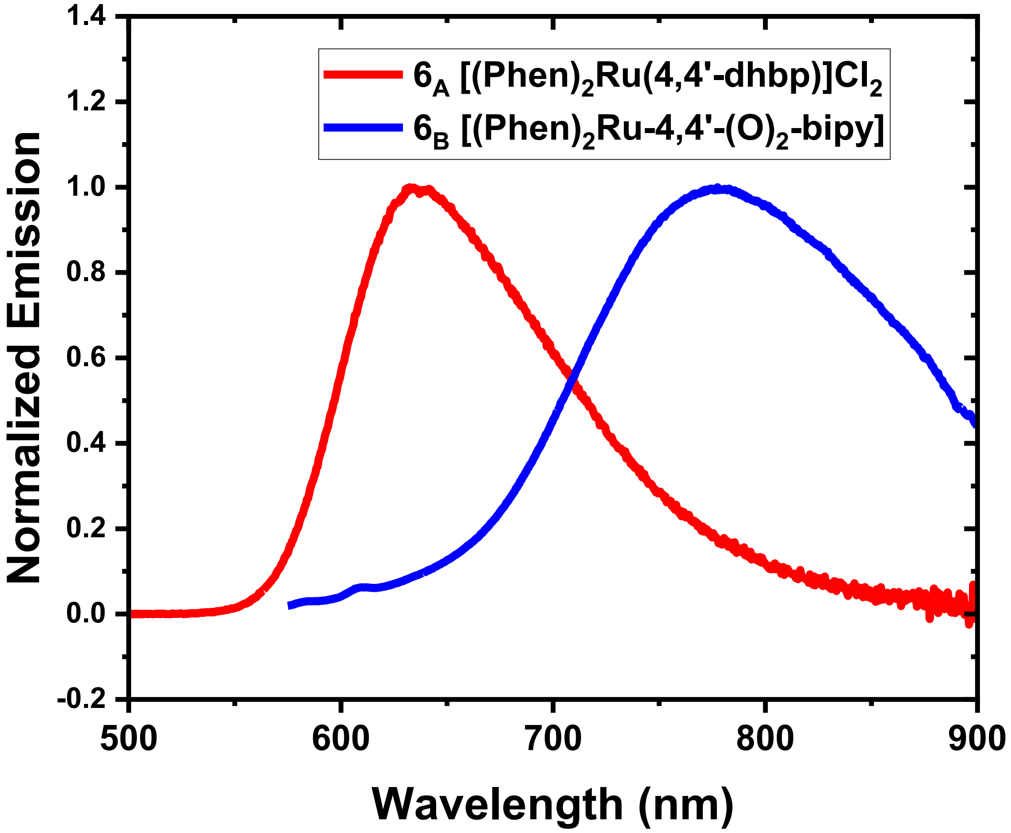

Figure S12: Room temperature overlaid photoluminescence spectra of 6_A and 6_B in acetonitrile

Complexes 7_A and 7_B

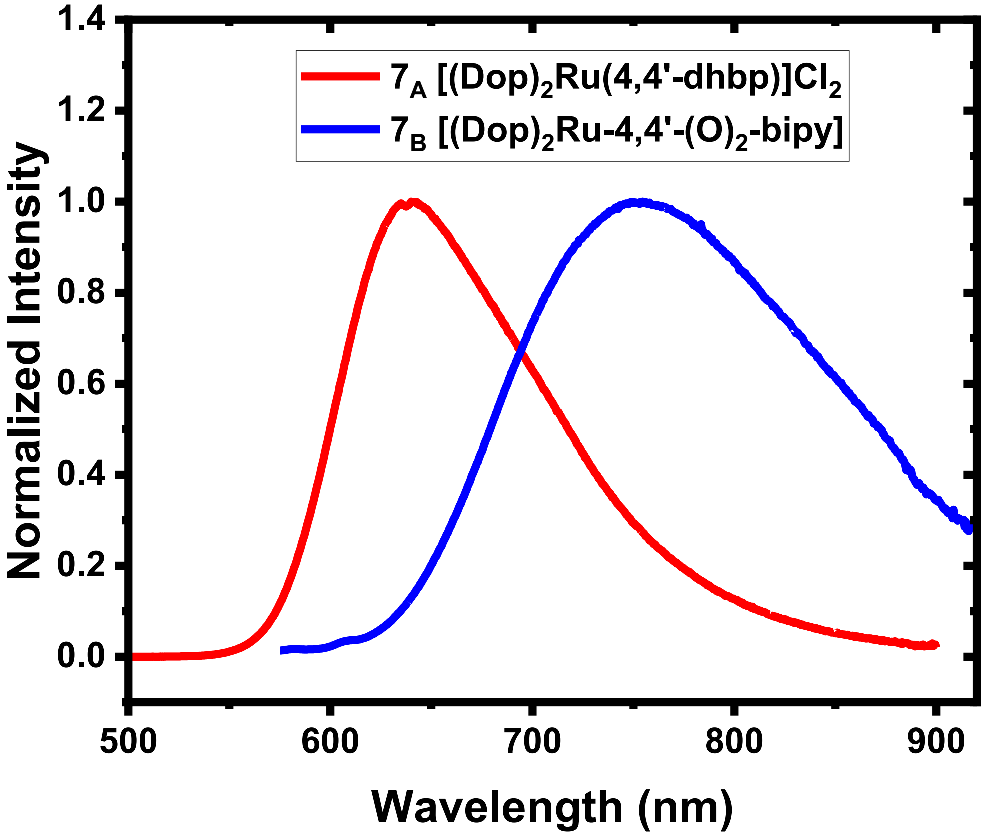

Figure S13: Room temperature overlaid photoluminescence spectra of 7_A and 7_B in acetonitrile

Photoluminescence (PL) Dynamics Studies

Complex 1_A

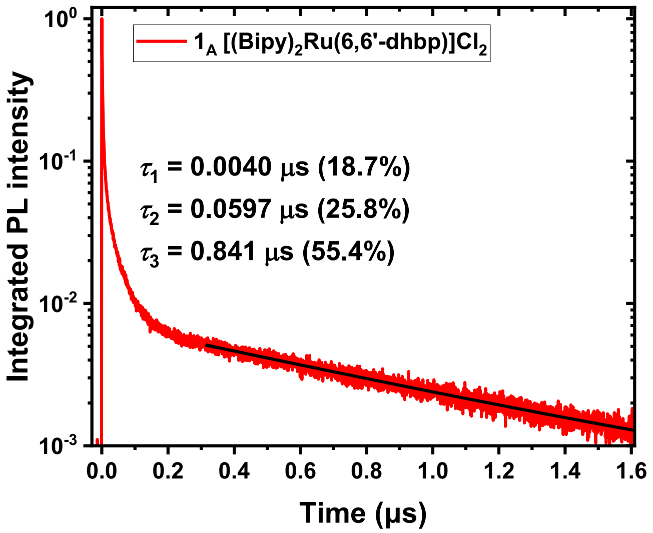

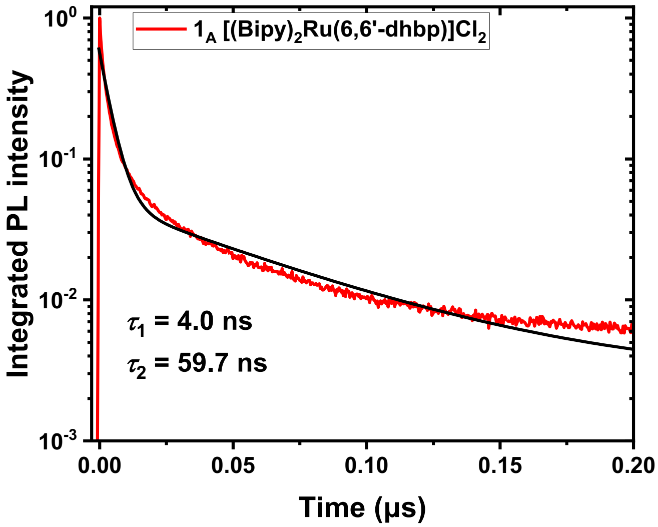

Figure S14. PL dynamics of $\mathbf{1_A}$ in deaerated acetonitrile excited at 404 nm. Top: full decay curve. Bottom: zoom in on the first 200 ns. Percentage indicates the fraction of all light that is emitted through the corresponding channel.

Complex 1_B

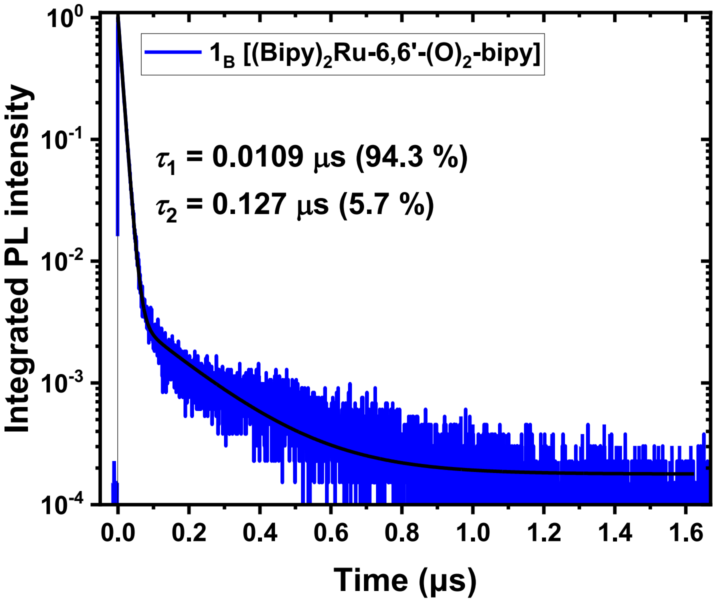

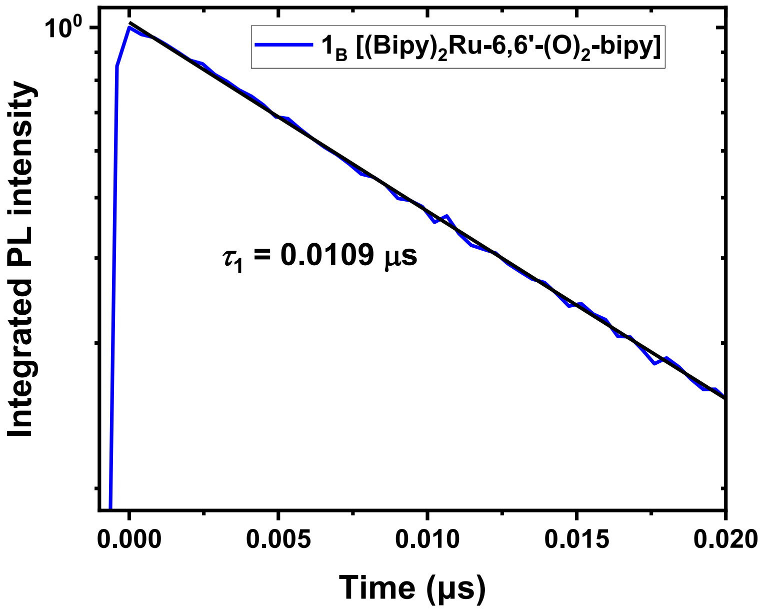

Figure S15. PL dynamics of $\mathbf{1_B}$ in deaerated acetonitrile excited at 404 nm. Top: full decay curve. Bottom: zoom in on the first 20 ns. Percentage indicates the fraction of all light that is emitted through the corresponding channel.

Complexes 2_A and 2_B

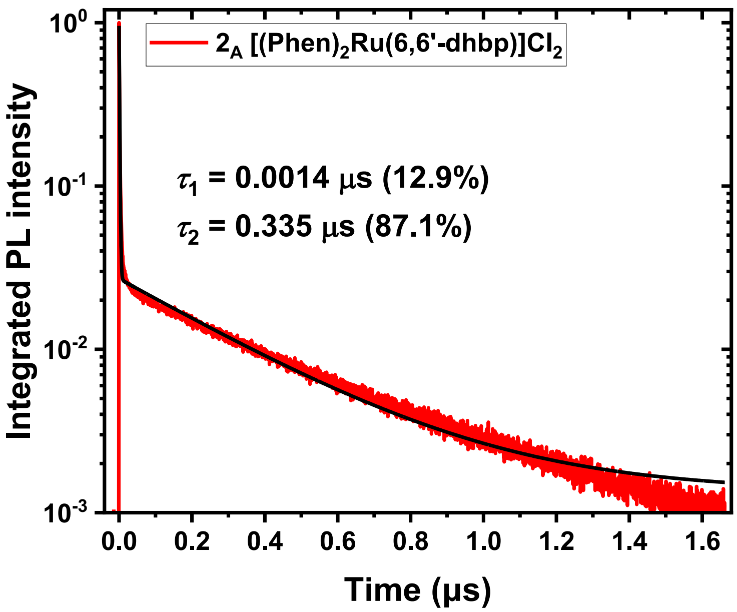

Figure S16. PL dynamics of $\mathbf{2_A}$ in deaerated acetonitrile excited at 404 nm. Percentage indicates the fraction of all light that is emitted through the corresponding channel.

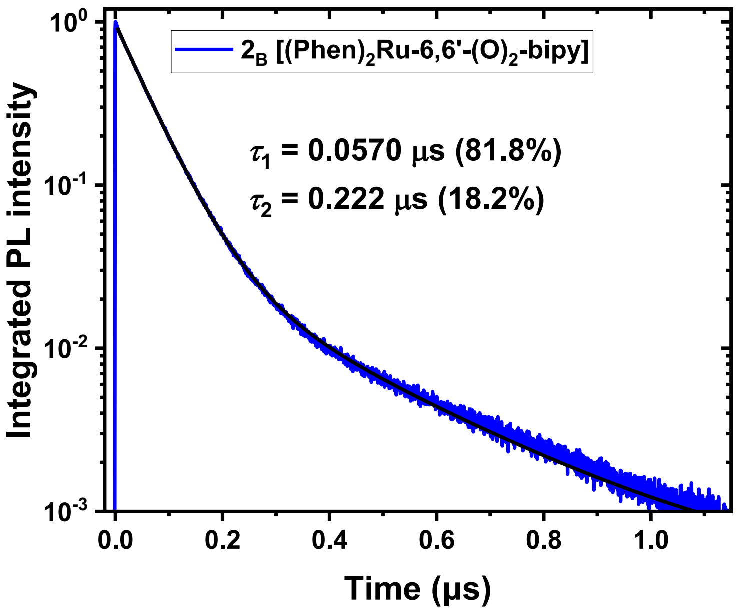

Figure S17. PL dynamics of $\mathbf{2_B}$ in deaerated acetonitrile excited at 404 nm. Percentage indicates the fraction of all light that is emitted through the corresponding channel.

Complexes 3_A and 3_B

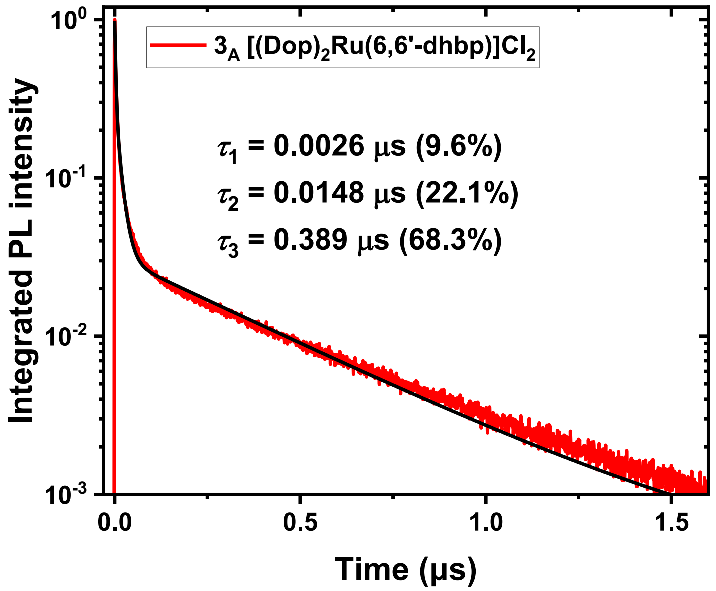

Figure S18. PL dynamics of $\mathbf{3_A}$ in deaerated acetonitrile excited at 404 nm. Percentage indicates the fraction of all light that is emitted through the corresponding channel.

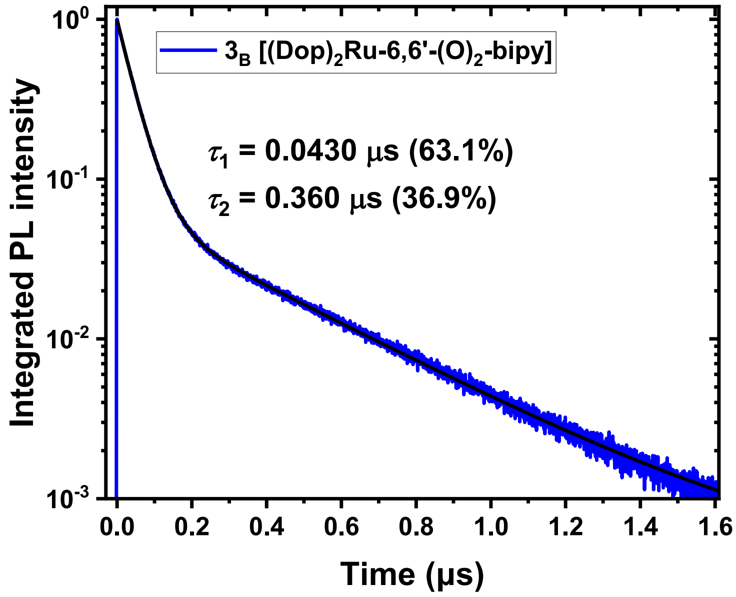

Figure S19. PL dynamics of $\mathbf{3_B}$ in deaerated acetonitrile excited at 404 nm. Percentage indicates the fraction of all light that is emitted through the corresponding channel.

Complexes 4_A and 4_B

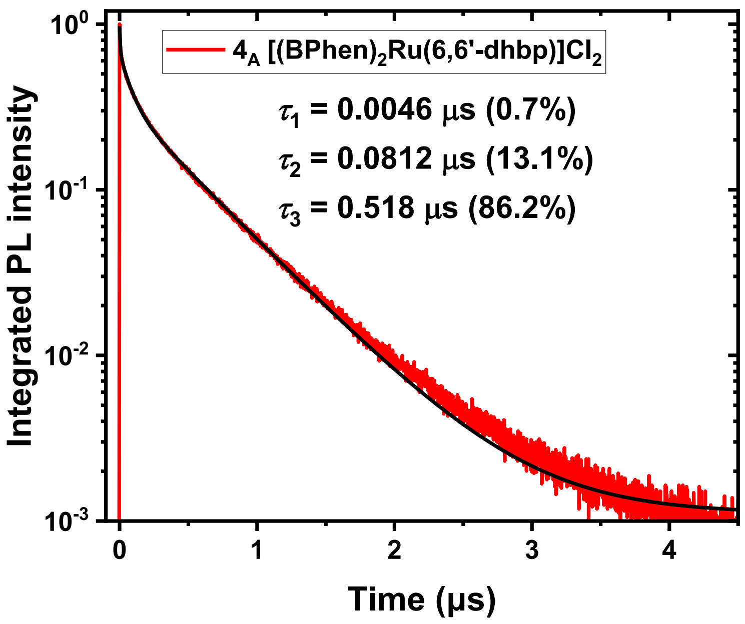

Figure S20. PL dynamics of $\mathbf{4_A}$ in deaerated acetonitrile excited at 404 nm. Percentage indicates the fraction of all light that is emitted through the corresponding channel.

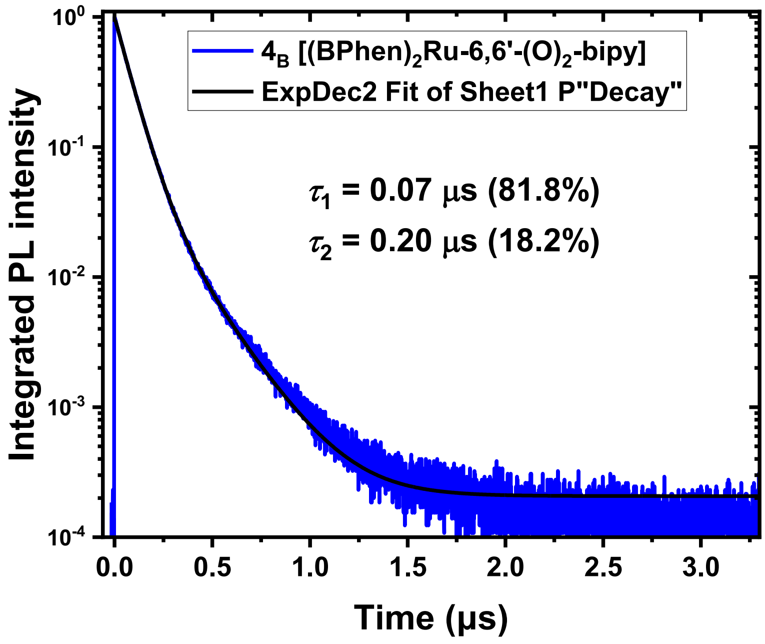

Figure S21. PL dynamics of $\mathbf{4_B}$ in deaerated acetonitrile excited at 404 nm. Percentage indicates the fraction of all light that is emitted through the corresponding channel.

Complexes 5_A and 5_B

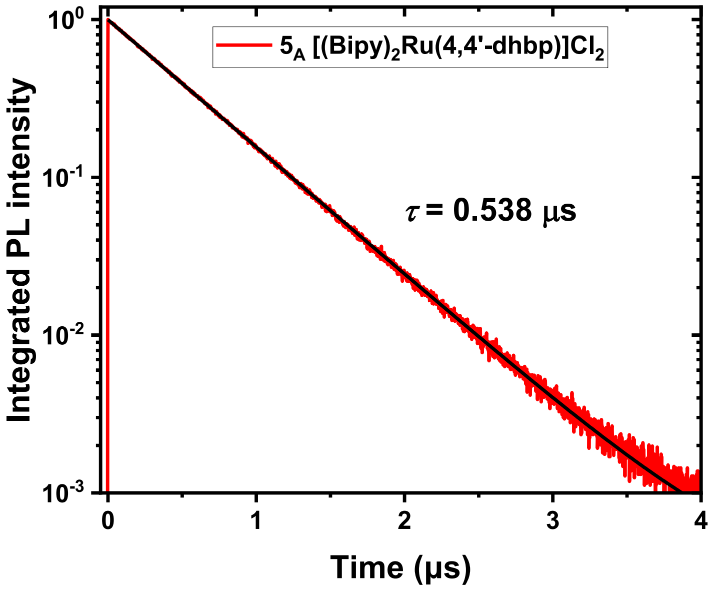

Figure S22. PL dynamics of $\mathbf{5_A}$ in deaerated acetonitrile excited at 404 nm.

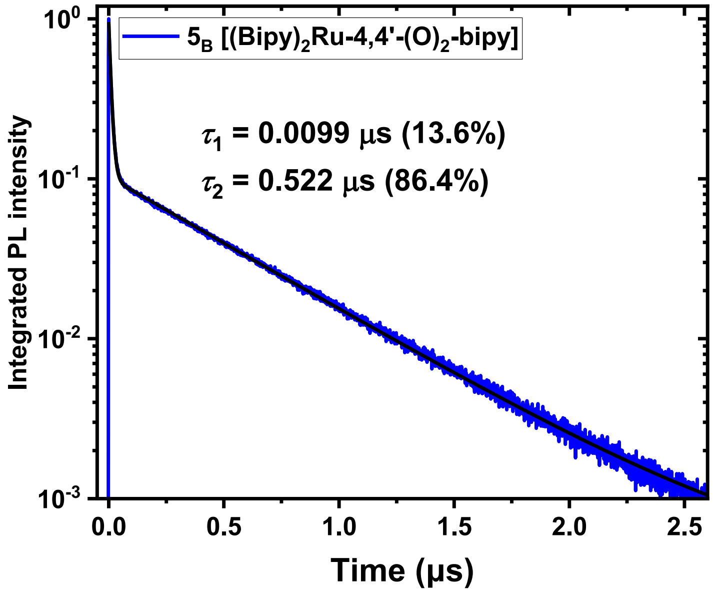

Figure S23. PL dynamics of $\mathbf{5_B}$ in deaerated acetonitrile excited at 404 nm. Percentage indicates the fraction of all light that is emitted through the corresponding channel.

Complexes 6_A and 6_B

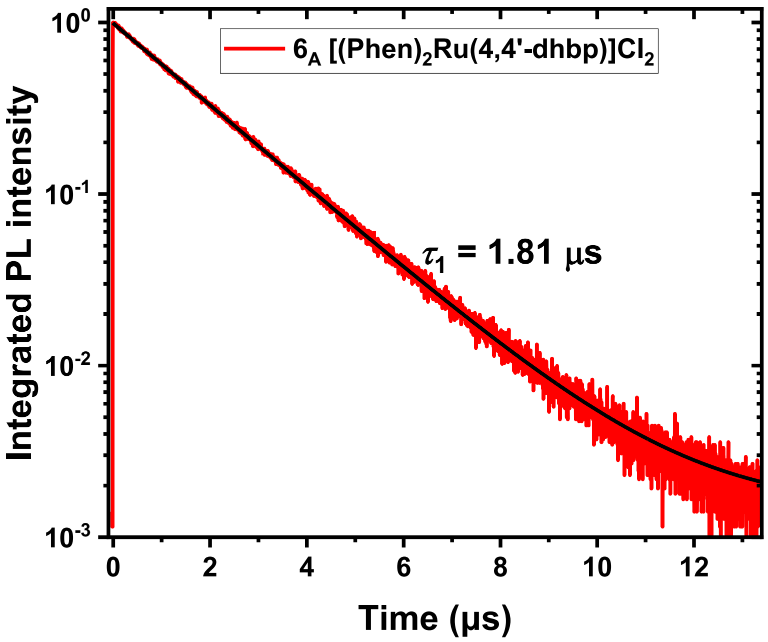

Figure S24. PL dynamics of $\mathbf{6_A}$ in deaerated acetonitrile excited at 404 nm.

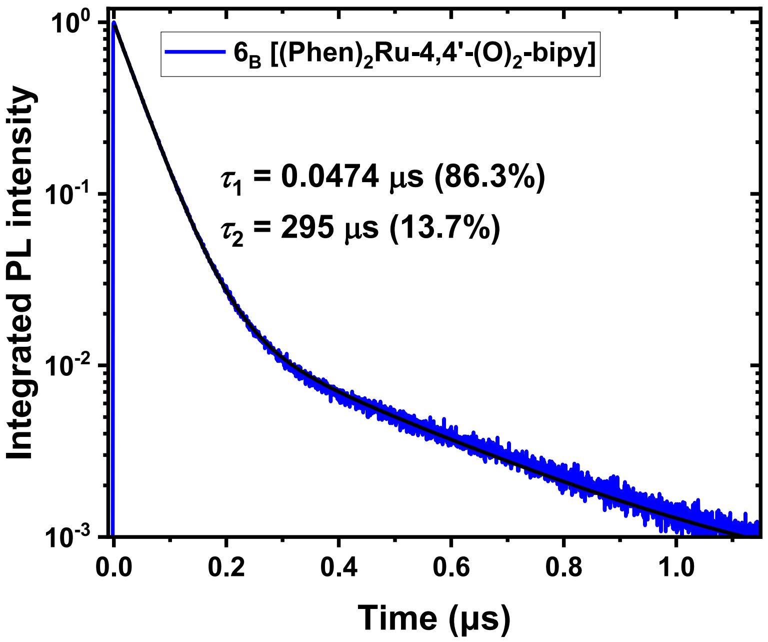

Figure S25. PL dynamics of $\mathbf{6_B}$ in deaerated acetonitrile excited at 404 nm. Percentage indicates the fraction of all light that is emitted through the corresponding channel.

Complexes 7_A and 7_B

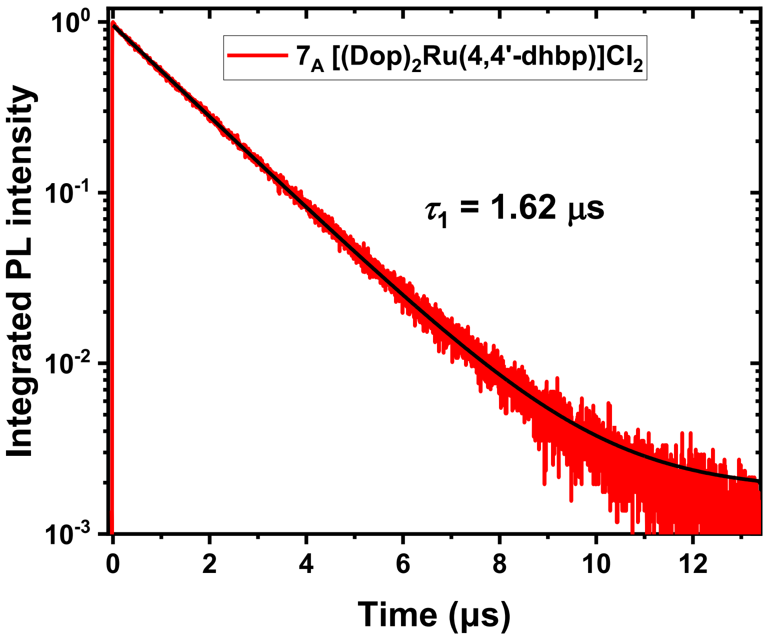

Figure S26. PL dynamics of 7_A in deaerated acetonitrile excited at 404 nm.

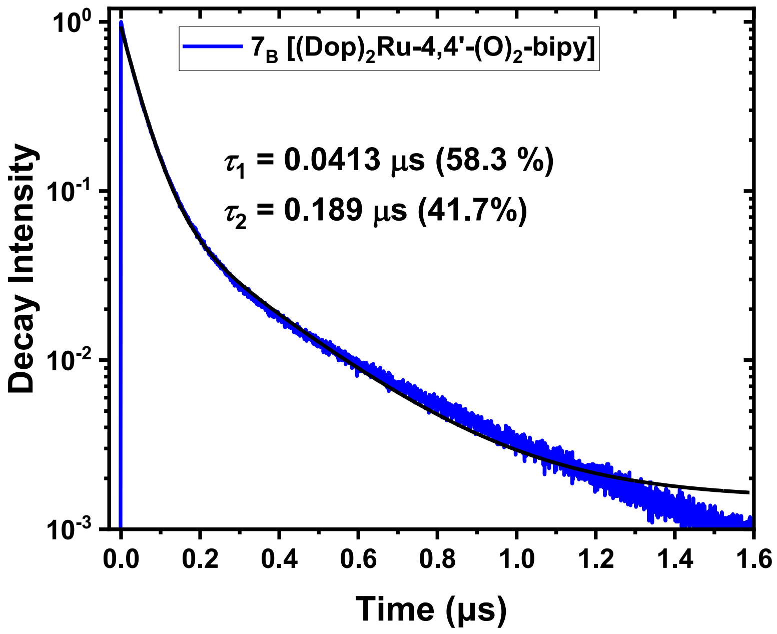

Figure S27. PL dynamics of 7_B in deaerated acetonitrile excited at 404 nm. Percentage indicates the fraction of all light that is emitted through the corresponding channel.

Complexes 8_A and 8_B

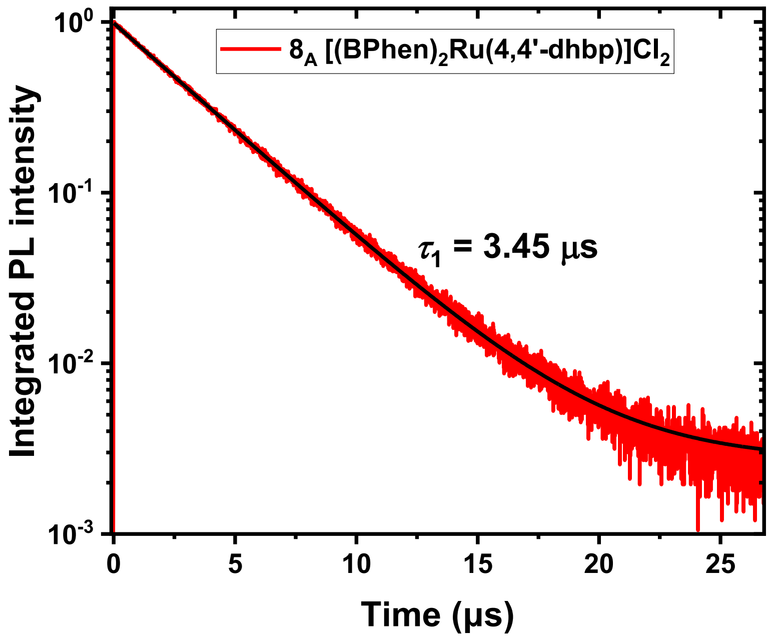

Figure S28. PL dynamics of $\mathbf{8_A}$ in deaerated acetonitrile excited at 404 nm.

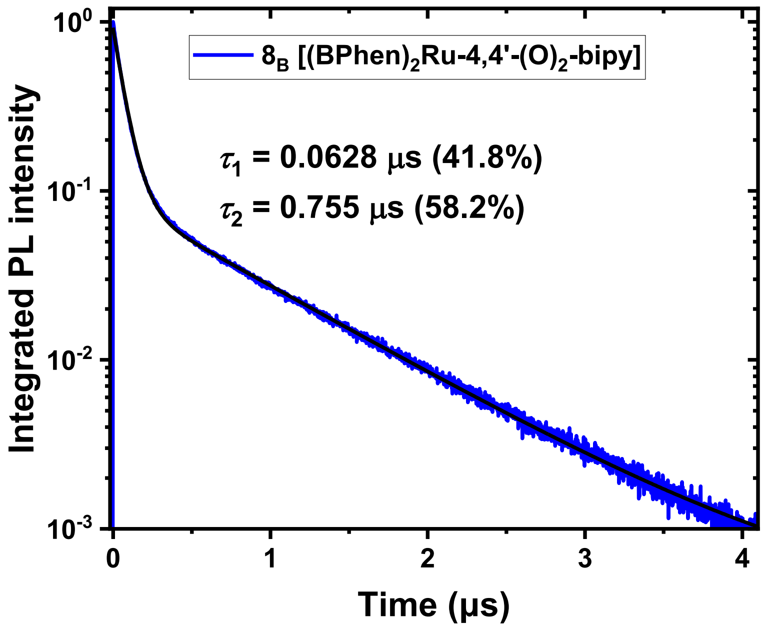

Figure S29. PL dynamics of $\mathbf{8_B}$ in deaerated acetonitrile excited at 404 nm. Percentage indicates the fraction of all light that is emitted through the corresponding channel.

Table S1. Excitation wavelengths, photoluminescence quantum yield, emission wavelengths, luminescence lifetimes and singlet oxygen quantum yields of compounds $[(bpy)_3Ru]Cl_2$, **1**$_A$-**8**$_A$ and **1**$_B$-**8**$_B$.

Compound name	λ max or Exc (nm)	ϕ_{Lum} (%)	λ_{Em} (nm)	Luminescence Lifetime(s) (τ) (μs)	Fraction of all light emitted through the corresponding channel
**$[(Bpy)_3Ru]Cl_2$**	451	9.4	617	0.882	100%
**1$_A$**	461	0.13	630	0.0040<br>0.0597<br>0.841	18.7%<br>25.8%<br>55.4%
**1$_B$**	544	0.04	727	0.0109<br>0.127	94.3%<br>5.7%
**2$_A$**	455	0.06	612	0.0014<br>0.335	12.9%<br>87.1%
**2$_B$**	517	0.29	710	0.0570<br>0.222	81.8%<br>18.2%
**3$_A$**	467	0.33	610	0.0026<br>0.0148<br>0.389	9.6%<br>22.1%<br>68.3%
**3$_B$**	529	0.34	734	0.0430<br>0.360	63.1%<br>36.9%
**4$_A$**	471	0.45	640	0.0046<br>0.0812<br>0.518	0.7%<br>13.1%<br>86.2%
**4$_B$**	548	0.17	760	0.0726<br>0.193	79.9%<br>20.1%
**5$_A$**	466	5.29	650	0.538	100%
**5$_B$**	527	0.58	775	0.0099<br>0.522	13.6%<br>86.4%
**6$_A$**	455	12.85	640	1.81	100%
**6$_B$**	515	0.25	745	0.0474<br>0.295	86.3%<br>13.7%
**7$_A$**	473	15.28	640	1.62	100%
**7$_B$**	528	0.087	763	0.0413<br>0.189	58.3%<br>41.7%
**8$_A$**	483	18.69	642	3.45	100%
**8$_B$**	525	0.5	704	0.0628<br>0.755	41.8%<br>58.2%

Table S2. Structural Parameters from the Single Crystal X-Ray Diffraction Data for $\mathbf{7_A}$.

**Compound**	$\mathbf{7_A}$
Formula	$C_{38}H_{34}Cl_2N_6O_9Ru$
$D_{calc.}$/ g cm^{-3}	1.599
μ/mm^{-1}	0.634
Formula Weight	890.68
Colour	dark red
Shape	block
Size/mm^3	0.15×0.10×0.07
T/K	100.01(10)
Crystal System	orthorhombic
Space Group	*Pbca*
a/Å	13.5194(2)
b/Å	18.9737(2)
c/Å	28.8521(4)
α/°	90
β/°	90
γ/°	90
V/Å^3	7400.94(17)
Z	8
Z'	1
Wavelength/Å	0.71073
Radiation type	MoK□
Θ_{min}/°	2.147
Θ_{max}/°	30.508
Measured Refl.	59664
Independent Refl.	11227
Reflections with I > 2(I)	8573
R_{int}	0.0371
Parameters	526
Restraints	0
Largest Peak	1.612
Deepest Hole	-1.256
GooF	1.032
wR_2 (all data)	0.1391
wR_2	0.1295
R_1 (all data)	0.0668
R_1	0.0478
